# n-Butanol production in *Saccharomyces cerevisiae* is limited by the availability of coenzyme A and cytosolic acetyl-CoA

**DOI:** 10.1186/s13068-016-0456-7

**Published:** 2016-02-24

**Authors:** Virginia Schadeweg, Eckhard Boles

**Affiliations:** Institute of Molecular Biosciences, Goethe-University Frankfurt, Max-von-Laue Str.9, 60438 Frankfurt Am Main, Germany

**Keywords:** n-butanol, *Saccharomyces*, ABE fermentation, acetyl-CoA, Coenzyme A, Pantothenate

## Abstract

**Background:**

Butanol isomers are regarded as more suitable fuel substitutes than bioethanol. n-Butanol is naturally produced by some *Clostridia* species, but due to inherent problems with clostridial fermentations, industrially more relevant organisms have been genetically engineered for n-butanol production. Although the yeast *Saccharomyces cerevisiae* holds significant advantages in terms of scalable industrial fermentation, n-butanol yields and titers obtained so far are only low.

**Results:**

Here we report a thorough analysis and significant improvements of n-butanol production from glucose with yeast via the acetoacetyl-CoA-derived pathway. First, we established an improved n-butanol pathway by testing various isoenzymes of different pathway reactions. This resulted in n-butanol titers around 15 mg/L in synthetic medium after 74 h. As the initial substrate of the n-butanol pathway is acetyl-coenzyme A (acetyl-CoA) and most intermediates are bound to coenzyme A (CoA), we increased CoA synthesis by overexpression of the pantothenate kinase *coaA* gene from *Escherichia coli*. Supplementation with pantothenate increased n-butanol production up to 34 mg/L. Additional reduction of ethanol formation by deletion of alcohol dehydrogenase genes *ADH1*-*5* led to n-butanol titers of 71 mg/L. Further expression of a mutant form of an ATP independent acetylating acetaldehyde dehydrogenase, adhE^A267T/E568K^, converting acetaldehyde into acetyl-CoA, resulted in 95 mg/L n-butanol. In the final strain, the n-butanol pathway genes, *coaA* and *adhE*^A267T/E568K^, were stably integrated into the yeast genome, thereby deleting another alcohol dehydrogenase gene, *ADH6*, and *GPD2*-encoding glycerol-3-phosphate dehydrogenase. This led to a further decrease in ethanol and glycerol by-product formation and elevated redox power in the form of NADH. With the addition of pantothenate, this strain produced n-butanol up to a titer of 130 ± 20 mg/L and a yield of 0.012 g/g glucose. These are the highest values reported so far for *S. cerevisiae* in synthetic medium via an acetoacetyl-CoA-derived n-butanol pathway.

**Conclusions:**

By gradually increasing substrate supply and redox power in the form of CoA, acetyl-CoA, and NADH, and decreasing ethanol and glycerol formation, we could stepwise increase n-butanol production in *S. cerevisiae*. However, still further bottlenecks in the n-butanol pathway must be deciphered and improved for industrially relevant n-butanol production levels.

**Electronic supplementary material:**

The online version of this article (doi:10.1186/s13068-016-0456-7) contains supplementary material, which is available to authorized users.

## Background

Biofuels produced by microbial fermentations of renewable resources represent an important replacement for fossil fuels. Today, bioethanol is the most important renewable gasoline substitute. However, higher alcohols like butanol are regarded as more suitable fuel substitutes, due to their higher energy density, lower hygroscopicity, and therefore lower corrosiveness [1].

n-Butanol is produced by various *Clostridium* species via the acetone butanol ethanol (ABE) fermentation process with a ratio of 1:3:6 and titers up to 13 g/l [2, 3]. However, clostridial fermentations are associated with several problems, including sporulation, slow growth rates, bacteriophage infections, and strict anaerobic cultivations [4]. For this reason, other industrially more relevant organisms have been engineered for n-butanol production by introducing variants of the clostridial pathway [5] or using ketoacid degradation pathways [6]. Although n-butanol titers up to 15 g/l [7] were achieved in *E. coli*, large-scale industrial fermentations with this bacterium also suffer from the high risk of phage infections and bacterial contaminations, for instance [8, 9]. Compared to *E. coli,S. cerevisiae* holds significant advantages in terms of scalable industrial fermentations, due to long-lasting experiences with this yeast in fermentation processes, high robustness, and tolerance against inhibitory compounds [10].

*S. cerevisiae* is able to produce small amounts of n-butanol on rich medium via endogenous pathways dependent on threonine or glycine catabolism, the first one stimulated by deletion of *ADH1* [11, 12]. By improving these pathways, n-butanol titers up to 242.8 and 92 mg/L, respectively, could be achieved. Combining the amino acid-dependent endogenous pathways with a synthetic ABE pathway and a mutant version of translation initiation factor eIF2B in an *adh1* mutant strain resulted in up to 300 mg/L n-butanol, but only in YEPD medium after 15–20 days [13]. Nevertheless, as biofuel production from amino acids is economically not viable, for industrial use threonine or glycine overproduction strains would be required which, however, exhibit lower theoretical maximum yields [1].

**Fig. 1:**
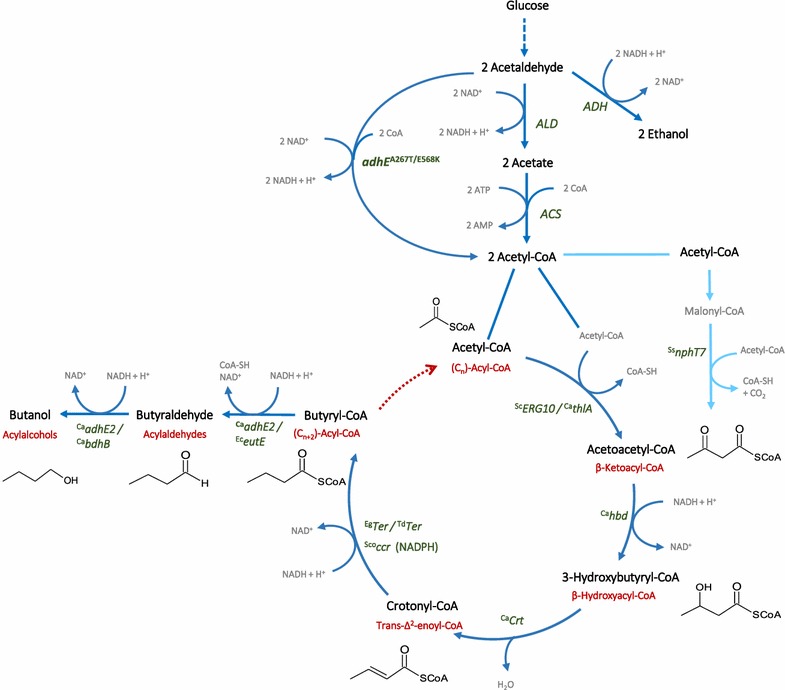
Metabolic pathway for n-butanol production via reverse β-oxidation in yeast. n-Butanol production and reverse β-oxidation are shown as a process of one or several rotations, respectively, of an acetoacetyl-CoA-derived synthesis pathway. This pathway can generate various compounds with different chain lengths. The different isoenzymes used in this study (Fig. 2, Table 2) are shown for each reaction. The reaction of ^Ss^ *nphT7* (use of malonyl-CoA and acetyl-CoA for production of acetoacetyl-CoA) is shown in *light blue*

Therefore, it is more promising to enhance n-butanol production by improvements of variants of the n-butanol pathway expressed in *S. cerevisiae*. In this pathway, two molecules of acetyl-CoA are condensed to acetoacetyl-CoA, which is reduced to 3-hydroxybutyryl-CoA and then dehydrated to crotonyl-CoA. This is further reduced to butyryl-CoA, butyraldehyde, and finally n-butanol (Fig. 1). Extending the pathway at the level of butyryl-CoA in a reversal of the β-oxidation cycle would enable even the production of longer molecules [14, 15]. Besides alcohols, also the intermediates of the pathway are valuable compounds or can be used to produce the corresponding aldehydes, fatty acids, and alkanes with different chain lengths. First attempts to establish n-butanol production in yeast using this acetoacetyl-CoA-derived pathway resulted in n-butanol titer as low as only 2.5 mg/L [16]. Further pathway optimization and engineering of substrate supply could enhance n-butanol titers up to 120 mg/L [9, 17]. However, these titers were obtained only in synthetic complete media in high-cell density fermentations and were critically reviewed in [1].

As n-butanol yield in yeast is still much lower than in *E. coli*, it was hypothesized that cytosolic acetyl-CoA as the initial substrate of the n-butanol pathway is a limiting factor [9]. In yeast, acetyl-CoA is present in four different compartments: cytosol, mitochondria, peroxisomes, and the nucleus. Cytosolic acetyl-CoA is produced via the decarboxylation of pyruvate into acetaldehyde, which is oxidized to acetate by aldehyde dehydrogenases and finally converted to acetyl-CoA, at the expense of two ATPs. This pathway is known as pyruvate dehydrogenase (Pdh) bypass. Aldehyde dehydrogenases compete with alcohol dehydrogenases (Adh) which due to the crabtree effect convert most of the sugar-derived acetaldehyde into ethanol [18]. On the other hand, mitochondrial acetyl-CoA obtained via pyruvate dehydrogenase obviously cannot be transported to the cytosol [19]. Different strategies were tested to increase the level of cytosolic acetyl-CoA. In one approach, alcohol dehydrogenases were deleted, the reactions of the Pdh bypass were overexpressed, and the withdrawal of acetyl-CoA into the glyoxylate cycle was prevented [18]. In another approach, an ATP independent pyruvate dehydrogenase complex from *Enterococcus faecalis* was expressed in the yeast cytosol replacing the Pdh bypass, but being dependent on supplementation with lipoic acid [20]. Another possibility of ATP independent cytosolic acetyl-CoA production is the use of acetylating acetaldehyde dehydrogenases or pyruvate formate lyases [21, 22].

Since the production of cytosolic acetyl-CoA proceeds from two substrates, acetate and coenzyme A (CoA), the level of CoA might limit acetyl-CoA production as well. Moreover, nearly all the intermediates of the n-butanol pathway exist as CoA esters which might additionally decrease CoA availability. Therefore, we hypothesized that engineering of CoA biosynthesis could further increase the concentration of acetyl-CoA in yeast, resulting in elevated n-butanol production. CoA biosynthesis starts from pantothenate. In yeast, pantothenate can be produced via an endogenous pathway starting from 2-ketoisovalerate, derived from the valine metabolic pathway, and spermine, derived from l-ornithine and methionine [23]. Besides, it can be taken up from the medium by the transporter Fen2 [24]. Pantothenate is then converted into 4-phosphopantothenate by pantothenate kinase Cab1, which is postulated to catalyze the rate-limiting step in CoA biosynthesis. Cysteine is incorporated by phosphopantothenate cysteine ligase Cab2, which is weakly glucose repressed like Cab1 [23]. The resulting product is finally converted into CoA in three further steps [25].

In the present study, we first established and improved n-butanol production in *S. cerevisiae* by testing different variants of the acetoacetyl-CoA-derived pathway. We then improved synthesis of CoA by overexpression of pantothenate kinase coaA from *E. coli*, which is not inhibited by acetyl-CoA [23], or by overexpression of the yeast Fen2 pantothenate transporter. The level of CoA could further be increased by adding pantothenate to the medium. By deletion of alcohol dehydrogenases and glycerol-3-phosphate dehydrogenase genes, we increased the availability of acetaldehyde and NADH as driving forces for the n-butanol pathway. Overexpression of a mutant form of an acetylating acetaldehyde dehydrogenase could further improve n-butanol production, resulting in n-butanol titers and yields of up to 130 ± 20 mg/L and 0.012 g/g glucose, respectively. These are the highest values ever reported for *S. cerevisiae* in synthetic medium via the acetoacetyl-CoA-derived pathway.

## Results and discussion

### Comparison of different variants of an acetoacetyl-CoA-derived n-butanol pathway in yeast

**Fig. 2:**
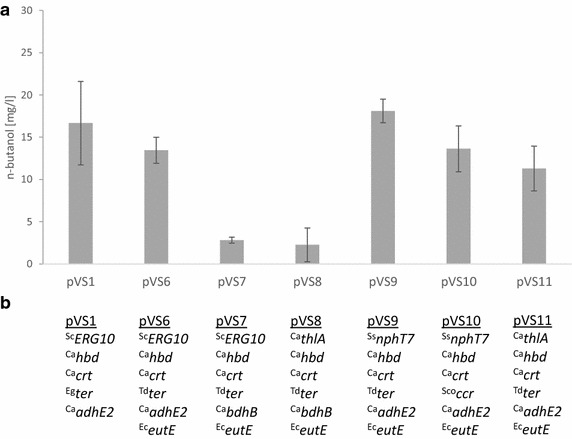
n-Butanol production with different variants of acetoacetyl-CoA-derived n-butanol pathways in *S. cerevisiae* strain CEN.PK113-5D. **a** The n-butanol concentrations after 74 h of semi-anaerobic fermentations in SMD medium are shown. *Error bars* represent the standard deviation of three independent replicates. **b** The respective enzyme combinations of n-butanol pathway vectors are displayed. Genes from *Saccharomyces cerevisiae* (Sc), *Clostridium acetobutylicum* (Ca), *E.coli* (Ec), *Euglena gracilis* (Eg), *Streptomyces collinus* (Sco), *Streptomyces sp*. CL190 (Ss), and *Treponema denticola* (Td) are indicated by prefixes in superscript

In order to engineer *S. cerevisiae* for n-butanol production, we introduced different variants of an acetoacetyl-CoA-derived n-butanol pathway in CEN.PK113-5D (Figs. 1, 2, Additional file 1: Figure S1). In our first experiments, we chose multicopy vectors for high-level expression of heterologous genes but soon recognized that the use of centromeric vectors generally resulted in significantly higher n-butanol titers (data not shown) and also higher enzyme activities of most of the pathway enzymes in crude extracts (Tables 1 and 2). Indeed, such problems by using multicopy vectors have also been reported by others [9, 20, 26]. The problems might be related to instabilities, vector burden, or transcriptional obstacles due to the high copy numbers and the strong promoters.

**Table 1:** Yeast strains and plasmids used in this study

Strain or plasmid	Characteristics	Reference
Strains		
CEN.PK113-5D	*MATa ura3*-*52 MAL2*-*8 c SUC2*	EUROSCARF, Frankfurt
VSY0	*MATa; ura3*-*52; trp1*-*289; leu2*-*3_112; his3*Δ*1; MAL2*-*8C; SUC2 adh1::loxP adh3::loxP adh5::loxP adh4*Δ*::loxP adh2*Δ*::LEU2*	This work
VSY8	*MATa; ura3*-*52; trp1*-*289; leu2*-*3_112; his3*Δ*1; MAL2*-*8C; SUC2 adh1::loxP adh3::loxP adh5::loxP adh4*Δ*::loxP adh2*Δ*::LEU2 sfa1*Δ: ^Ec^ *adhE* ^A267T/E568K^ *, hphNT1*	This work
VSY9	*MATa; ura3*-*52; trp1*-*289; leu2*-*3_112; his3*Δ*1; MAL2*-*8C; SUC2 adh1::loxP adh3::loxP adh5::loxP adh4*Δ*::loxP adh2*Δ*::LEU2 sfa1*Δ: ^Ec^ *adhE* ^A267T/E568K^ *, hphNT1 adh6*Δ: ^Ec^ *coaA, natNT2*	This work
VSY10	*MATa; ura3*-*52; trp1*-*289; leu2*-*3_112; his3*Δ*1; MAL2*-*8C; SUC2 adh1::loxP adh3::loxP adh5::loxP adh4*Δ*::loxP adh2*Δ*::LEU2 sfa1*Δ: ^Ec^ *adhE* ^A267T/E568K^ *, hphNT1 adh6*Δ: ^Ec^ *coaA, natNT2* *gpd2*Δ: ^Sc^ *ERG10,* ^Ca^ *hbd,* ^Ca^ *crt,* ^Td^ *ter,* ^Ca^ *adhE2,* ^Ec^ *eutE, kanMX*	This work
Plasmids		
pRS41K	CEN6ARS4*, kanMX, Amp* ^*r*^	[43]
pRS62K	2µ*, kanMX, Amp* ^*r*^ *, shortened HXT7 promoter and CYC1 terminator*	[44]
pRS62N	2µ, *natNT2, Amp* ^*r*^ *, shortened HXT7 promoter and CYC1 terminator*	[44]
pVS1_high copy	2µ*, kanMX, Amp* ^*r*^, reverse β-oxidation: ^Sc^ *ERG10,* ^Ca^ *hbd,* ^Ca^ *crt,* ^Eg^ *ter,* ^Ec^ *adhE2*	This work
pVS1	CEN6ARS4*, kanMX, Amp* ^*r*^, reverse β-oxidation: ^Sc^ *ERG10,* ^Ca^ *hbd,* ^Ca^ *crt,* ^Eg^ *ter,* ^Ca^ *adhE2*	This work
pVS4	CEN6ARS4, *hphNT1*, Amp^r^, ^Ec^ *adhE* ^A267T/E568K^	This work
pVS6_high copy	2µ, *kanMX, Amp* ^*r*^, reverse β-oxidation: ^Sc^ *ERG10,* ^Ca^ *hbd,* ^Ca^ *crt,* ^Td^ *ter,* ^Ec^ *eutE,* ^Ca^ *adhE2*	This work
pVS6	CEN6ARS4, *kanMX, Amp* ^*r*^, reverse β-oxidation: ^Sc^ *ERG10,* ^Ca^ *hbd,* ^Ca^ *crt,* ^Td^ *ter,* ^Ec^ *eutE,* ^Ca^ *adhE2*	This work
pVS7_high copy	2µ, *kanMX, Amp* ^*r*^, reverse β-oxidation: *ERG10*, ^Ca^ *hbd,* ^Ca^ *crt,* ^Td^ *ter,* ^Ec^ *eutE,* ^Ca^ *bdhB*	This work
pVS7	CEN6ARS4, *kanMX, Amp* ^*r*^, reverse β-oxidation: ^Sc^ *ERG10*, ^Ca^ *hbd,* ^Ca^ *crt,* ^Td^ *ter,* ^Ec^ *eutE,* ^Ca^ *bdhB*	This work
pVS8_high copy	2µ, *kanMX, Amp* ^*r*^, reverse β-oxidation: ^Ca^ *thlA,* ^Ca^ *hbd,* ^Ca^ *crt,* ^Td^ *ter,* ^Ec^ *eutE,* ^Ca^ *bdhB*	This work
pVS8	CEN6ARS4, *kanMX, Amp* ^*r*^, reverse β-oxidation: ^Ca^ *thlA,* ^Ca^ *hbd,* ^Ca^ *crt,* ^Td^ *ter,* ^Ec^ *eutE,* ^Ca^ *bdhB*	This work
pVS9	CEN6ARS4, *kanMX, Amp* ^*r*^, reverse β-oxidation: ^Ss^ *nphT7,* ^Ca^ *hbd,* ^Ca^ *crt,* ^Td^ *ter,* ^Ec^ *eutE,* ^Ca^ *adhE2*	This work
pVS10	CEN6ARS4, *kanMX, Amp* ^*r*^, reverse β-oxidation: ^Ss^ *nphT7,* ^Ca^ *hbd,* ^Ca^ *crt,* ^Sco^ *ccr,* ^Ec^ *eutE,* ^Ca^ *adhE2*	This work
pVS11	CEN6ARS4, *kanMX, Amp* ^*r*^, reverse β-oxidation: ^Ca^ *thlA,* ^Ca^ *hbd,* ^Ca^ *crt,* ^Td^ *ter,* ^Ec^ *eutE,* ^Ca^ *adhE2*	This work
pVS5_1	2µ, *natNT2,* Amp^r^, CoA increase: ^Sc^ *FEN2*, pMET25-^Ec^ *coaA*	This work
pVS5_2	2µ, *natNT2*, Amp^r^, CoA increase: ^Sc^ *FEN2*, ^Sc^ *CAB2*	This work
pVS5_3	2µ, *natNT2*, Amp^r^, CoA increase: ^Sc^ *FEN2*	This work
pVS5_4	2µ, *natNT2*, Amp^r^, CoA increase: pMET25-^Ec^ *coaA*	This work
pVS5_5	2µ, *natNT2*, Amp^r^, CoA increase: ^Ec^ *coaA*	This work
pVS5_6	2µ, *natNT2*, Amp^r^, CoA increase: pMET25-^Sc^ *FEN2*	This work

Genes from *Saccharomyces cerevisiae* (Sc), *Clostridium acetobutylicum* (Ca), *E.coli* (Ec), *Euglena gracilis* (Eg), *Streptomyces collinus* (Sco), *Streptomyces sp*. CL190 (Ss), and *Treponema denticola* (Td) are indicated by prefixes in superscript. The *MET25* promoter is indicated as “pMET25”; other promoters are indicated in Additional file 2: Table S1

*kanMX* G418 resistance, *hphNT1* hygromycin resistance, *natNT2* nourseothricin resistance, *Amp* ^*r*^ ampicillin resistance

**Table 2:** In vitro activities of various enzymes involved in n-butanol production pathways expressed in *S. cerevisiae*

Enzyme	Activity [U/mg] high copy vector	Activity [U/mg] low copy vector
Thiolase		
^Sc^ *ERG10*	0.043 ± 0.009	0.10 ± 0.005
^Ca^ *thlA*	0.15 ± 0.04	4.96 ± 0.4
None	0.003 ± 0.0003	0.004 ± 0.002
Acetyl-CoA:malonyl-CoA acyltransferase		
^Ss^ *nphT7*	NM	0.12 ± 0.009
None	NM	0.002 ± 0.001
3-Hydroxybutyryl-CoA DH		
^Ca^ *hbd*	0.31 ± 0.04	1.47 ± 0.19
None	0.011 ± 0.004	0.063 ± 0.01
Crotonase		
^Ca^ *crt*	3.5 ± 1.07	35.64 ± 3.2
None	0.17 ± 0.04	4.09 ± 2.2
Trans-2-enoyl-CoA reductase		
^Eg^ *ter*	ND	0.002 ± 0.001
^Td^ *ter*	ND	0.0103 ± 0.002
None	ND	ND
Crotonyl-CoA reductase		
^Sco^ *ccr*	NM	0.001 ± 0.00009
None	NM	ND
Butyraldehyde-DH		
^Ca^ *adhE2* (pVS1)	0.00027 ± 0.00002	0.0039 ± 0.003
^Ca^ *adhE2* + ^Ec^ *eutE* (pVS6)	0.00091 ± 0.0004	0.06 ± 0.009
^Ec^ *eutE*	0.014 ± 0.0083	0.044 ± 0.004
None	ND	0.0022 ± 0.0009
Butanol-DH		
^Ca^ *adhE2* (pVS1)	0.07 ± 0.02	0.062 ± 0.015
^Ca^ *adhE2* (pVS6)	0.053 ± 0.02	0.076 ± 0.005
^Ca^ *bdhB*	0.073 ± 0.03	0.044 ± 0.008
None	0.049 ± 0.01	0.04 ± 0.009

The mean values of three independent replicates are shown with standard deviations. One unit (U) is defined as the conversion of 1 µmol of substrate into the corresponding product per 1 min. The term “none” refers to the empty vector control, which means for the high copy variants (2 µ) pRS62K and for the low copy variants (CEN6/ARS4) pRS41K. Genes from *Saccharomyces cerevisiae* (Sc), *Clostridium acetobutylicum* (Ca), *E.coli* (Ec), *Euglena gracilis* (Eg), *Streptomyces collinus* (Sco), *Streptomyces sp*. CL190 (Ss), and *Treponema denticola* (Td) are indicated by prefixes in superscript

*ND* not detectable, *NM* not measured

On the other hand, even without a heterologous pathway, *adh1* mutants of *S. cerevisiae* are able to produce n-butanol in YEPD medium via an endogenous 2-keto acid-derived pathway from threonine [11]. As we observed comparable results in YEPD medium with Δ*adh1*-*5* (VSY0) and Δ*adh1,3,5* (JDY4) mutant strains (n-butanol production from 50 mg/L up to 120 mg/L; data not shown), all further fermentations were carried out in synthetic minimal medium with 20 g/L glucose (SMD), where no n-butanol production was observed for VSY0 (Additional file 1: Figure S2). For most reactions of the acetoacetyl-CoA-derived pathway, we tested several isoenzymes from different organisms. We performed fermentation experiments (Fig. 2, Additional file 1: Figure S1) and also tested enzyme activities in crude extracts (Table 2). We tested the different isoforms in various combinations, always combined on a single centromeric plasmid (Table 1). Several strong promoters and terminators were used for expression (Additional file 2: Table S1). Combinations were chosen due to the measured enzyme activities, and enzymes were also changed if they were assumed to catalyze critical steps.

All fermentations were started with an OD_600_ of 0.3 and were performed for at least 100 h under semi-anaerobic conditions at 30 °C. The highest n-butanol titers were reached at about 74 h with close to 20 mg/L (Additional file 1: Figure S1). The gene combinations on plasmids pVS1, 6, 9, 10, and 11 turned out to perform best (Fig. 2). The bad performance of pVS7 and pVS8 seems to be due to the low activity of butanol dehydrogenase encoded by *bdhB* [27]. For the thiolase reaction, the yeast Erg10 enzyme and the *C. acetobutylicum* thlA resulted in comparable n-butanol production although the in vitro activity of the bacterial enzyme seems to be considerably higher. However, the enzyme assay is performed in the reverse reaction by conversion of acetoacetyl-CoA into two acetyl-CoA. NphT7 as a malonyl-CoA-dependent acyltransferase, which catalyzes an irreversible reaction [28], only marginally improved n-butanol production but works on the expense of one additional ATP for malonyl-CoA synthesis. For the conversion of crotonyl-CoA to butyryl-CoA, the trans-2-enoyl-CoA reductase ter enzymes [9] seem to be superior to crotonyl-CoA reductase ccr [29] (compare pVS10 and pVS9). Although the butyraldehyde dehydrogenase eutE [15] did not improve n-butanol production, assuming that the different ter enzymes did not have a great influence, we decided to include it in our further experiments due to its high enzyme activity and more reliable n-butanol production (see low standard deviation). Therefore, we continued with the enzyme combinations of pVS6, because of acetyl-CoA-dependent yeast’s own thiolase (Erg10) reaction, which is superior to ATP- and malonyl-CoA-consuming NphT7 of pVS9.

## Overexpression of pantothenate kinase coaA and addition of pantothenate increase n-butanol production

**Fig. 3:**
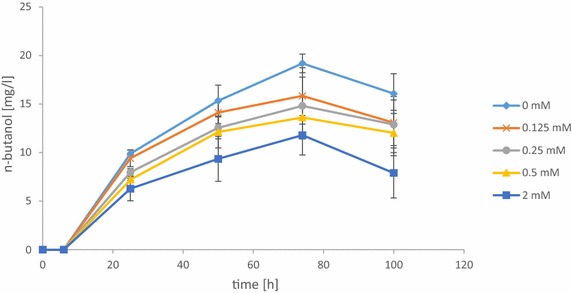
n-Butanol production with n-butanol pathway (pVS6) and *coaA* overexpression under control of the *MET25* promoter (pVS5_4) in CEN.PK113-5D with addition of methionine. Five different concentrations of methionine were added to SMD medium: 0 mM (*diamond*), 0.125 mM (*cross*), 0.25 mM (*circle*), 0.5 mM (*triangle*), and 2 mM (*square*). *Error bars* represent the standard deviation of three independent replicates

Although we could demonstrate reasonable n-butanol production from glucose with the heterologous acetoacetyl-CoA-derived pathways, the yields and titers were still low. As the substrate acetyl-CoA and also most intermediates of the pathway are bound to coenzyme A (CoA), we speculated that a limiting availability of CoA might be a reason for the low n-butanol production. Therefore, we aimed to increase the CoA pool by optimization of its anabolic synthesis route. In yeast, CoA can either be produced via the endogenous pantothenate synthesis pathway [23] or from exogenously supplied pantothenate. We overexpressed pantothenate kinase as this step has been reported to limit CoA synthesis [25]. We chose the coaA enzyme from *E. coli* as this is not inhibited by acetyl-CoA [30]. On the other hand, we overexpressed the yeast pantothenate transporter Fen2. *CoaA* and *FEN2* were expressed under control of the methionine-repressible *MET25* promoter [31]. The *MET25* promoter is most active in the absence of methionine and is gradually repressed up to 1 mM methionine, whereas 2 mM as a final concentration was chosen to guarantee repression. Therefore, fermentations were performed in SMD medium with strain CEN.PK113-5D transformed with pVS6 (n-butanol pathway) and either pVS5_4 (*coaA*) or pVS5_6 (*FEN2*), in the presence of different amounts of methionine. With decreasing methionine concentrations and therefore increasing expression of *coaA* or *FEN2*, in both cases the production of n-butanol increased up to 19 and 16 mg/L, respectively (Fig. 3, Additional file 1: Figure S3). Actually, the SMD medium contains 0.84 µM pantothenate originating from the yeast nitrogen base (BD Difco YNB). Surprisingly, additional overexpression of phosphopantothenoylcysteine synthetase Cab2 or co-overexpression of coaA and Fen2 led to a decrease in n-butanol production (data not shown). For unknown reasons, it was not possible to get yeast transformants which overexpress all three genes together, even when expressed behind other promoters. Nevertheless, the results indicate that the availability of CoA is indeed a limiting factor for n-butanol production. However, it might be that also other CoA-derived products will increase. But, as we overexpress acetylating acetaldehyde dehydrogenase, which needs CoA for acetyl-CoA synthesis, most of the CoA will increase acetyl-CoA production. Furthermore, a lot of CoA is bound to the intermediates of n-butanol pathway and is not available for other reactions.

**Fig. 4:**
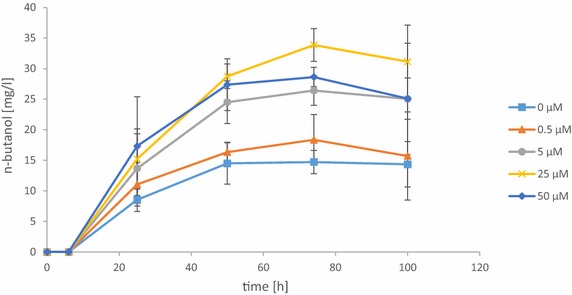
n-Butanol production with n-butanol pathway (pVS6) and *coaA* overexpression (pVS5_4) in CEN.PK113-5D with addition of pantothenate. Five different concentrations of pantothenate were added to SMD medium: 0 µM (*square*), 0.5 µM (*triangle*), 5 µM (*circle*), 25 µM (*cross*), and 50 µM (*diamond*). *Error bars* represent the standard deviation of three independent replicates

To investigate whether higher concentrations of pantothenate can further increase n-butanol production, fermentations were performed with the *coaA*-overexpressing strain CEN.PK113-5D (pVS6, pVS5_4) after addition of 0–50 µM pantothenate to SMD medium (without methionine). n-Butanol production further increased from 15 mg/L (0 µM pantothenate) to 34 mg/L at a pantothenate concentration of 25 µM but decreased slightly at 50 µM pantothenate (Fig. 4). Nevertheless, for industrial applications pantothenate addition would be too expensive and not acceptable. Therefore, our results indicate that for industrial settings it might be promising to increase flux through the endogenous pantothenate synthesis pathway together with coaA overexpression.

**Fig. 5:**
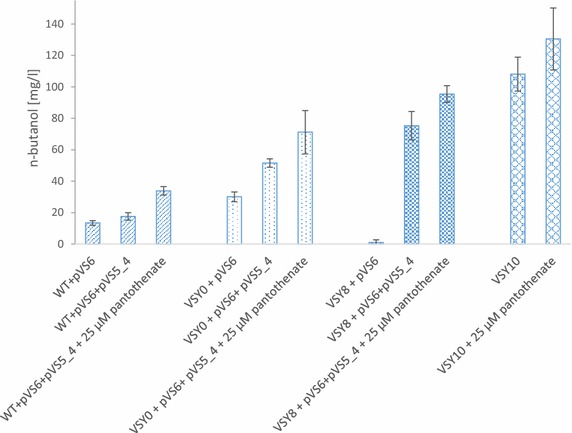
Comparison of n-butanol production with different strains. n-Butanol concentrations after 74 h of semi-anaerobic fermentations in SMD medium are shown. Compared are the fermentations of the wildtype CEN.PK113-5D, VSY0 (Δ*adh1*-*5*), and VSY8 (Δ*adh1*-*5 sfa1* with *adhE* ^A267T/E568K^) with plasmid pVS6 (n-butanol pathway), with or without *coaA* overexpression (pVS5_4), and strain VSY10 (Δ*adh1*-*6 sfa1 gpd2,* with n-butanol pathway genes of pVS6, *coaA*, and *adhE* ^A267T/E568K^), in the absence or presence of additional 25 µM pantothenate, in SMD medium. *Error bars* represent the standard deviation of three independent replicates and a statistical analysis is shown in Additional file 2: Table S4

For all important fermentations in this study, n-butanol titers (mg/L) are compared in Fig. 5, n-butanol yields (g/g consumed glucose) in Additional file 1: Figure S4, glucose consumption/residual glucose in Additional file 1: Figure S5, ethanol and glycerol production in Additional file 1: Figure S6, and OD_600_ values in Additional file 1: Figure S7.

## Deletion of alcohol dehydrogenases creates a push to n-butanol production

The next step was to redirect the metabolic flux away from the predominant fermentation product ethanol to n-butanol, and to increase the redox power in the form of NADH. Therefore, alcohol dehydrogenase genes *ADH1*-*5* were deleted in order to reduce NADH-dependent acetaldehyde reduction and the concomitant formation of ethanol. As expected, we observed less ethanol production, but therefore an increased glycerol formation as a result of an enhanced alternative NADH oxidation route of yeast (Additional file 1: Figure S6). Fermentations of VSY0 (Δ*adh1*-*5*) transformed with pVS6 (n-butanol pathway) resulted in n-butanol production up to 30 mg/L (Additional file 1: Figure S2), which is a significant increase compared to the reference strain (13 mg/L) (Fig. 5). The effect of elevated CoA supply was also tested in this strain by introducing pVS5_4 (*coaA*) together with pVS6. n-Butanol production was significantly higher in the *adh1*-*5* deletion strain with *coaA* overexpression (52 versus 30 mg/L) (Additional file 1: Figure S2, Fig. 5). After the addition of 25 µM pantothenate, the final n-butanol titer even increased up to 71 mg/L in the Δ*adh1*-*5* (pVS6, pVS5_4) strain (Additional file 1: Figure S8, Fig. 5). These results show that providing more substrate in the form of acetaldehyde and more reducing power in the form of NADH can increase n-butanol production.

## Improving n-butanol production with an acetylating acetaldehyde dehydrogenase

In yeast, cytosolic acetyl-CoA is mainly produced by acetyl-CoA synthetases Acs1 and 2 [18]. It has been shown before that acetyl-CoA-dependent product formation can be increased by increasing acetyl-CoA formation [8, 32]. However, acetyl-CoA synthetases use hydrolysis of ATP to AMP which is not favorable for anaerobic n-butanol production, since only glycolysis and not respiration can provide ATP. Therefore, we tested whether the expression of an ATP independent acetylating acetaldehyde dehydrogenase from *E. coli*, *adhE*, can further increase n-butanol production. Normally, the enzyme adhE catalyzes the reduction of acetyl-CoA into ethanol. Membrillo-Hernandez and coworkers [33] succeeded in engineering the enzyme to mainly perform the reverse reaction of converting acetaldehyde into acetyl-CoA. The specific acetaldehyde dehydrogenase activity could be increased by changing two amino acids: A267T and E568 K. For this reason, we chose to use adhE^A267T/E568K^ to increase the cytosolic acetyl-CoA pool. The mutant *adhE*^A267T/E568K^ allele could complement the growth defect of an *acs2* mutant strain on glucose medium, indicating that it is functional in yeast (data not shown) [21, 22]. We integrated this allele, expressed under control of the strong glycolytic *PFK1* promoter, into the *SFA1* locus resulting in strain VSY8. As *SFA1* encodes an additional but minor alcohol dehydrogenase [34], we chose this locus for integration to stabilize the strain and to prevent the occurrence of ethanol-producing suppressor mutants. Surprisingly, fermentations of VSY8 expressing the n-butanol pathway (pVS6) showed almost no n-butanol production, but increased ethanol production instead (Fig. 5, Additional file 1: Figure S6). One reason could be that adhE^A267T/E568K^ mainly converts acetaldehyde or acetyl-CoA into ethanol if there is not enough free CoA, the second substrate for the acetylating acetaldehyde dehydrogenase activity, available. Indeed, concomitant overexpression of *coaA* reduced ethanol and increased n-butanol formation (Additional file 1: Figure S9, Additional file 1: Figure S6). On the other hand, it might be possible that the additional alcohol dehydrogenase gene *ADH6* is upregulated under those conditions. Indeed, when *ADH6* was deleted by integration of *coaA*, a further decrease in ethanol formation could be observed [calculated yield for ethanol: 0.1 g/g glucose in VSY10 and 0.13 g/g in VSY8 (pVS6, pVS5_4)] (Additional file 1: Figure S6). In fermentations of the Δ*adh1*-*5*, *adhE*^A267T/E568K^-overexpressing strain VSY8 (pVS6, pVS5_4), n-butanol production was increased up to 75 mg/L, which is a significant improvement compared to the strain without *adhE*^A267T/E568K^ overexpression (52 mg/L) (Fig. 5). To further increase CoA availability, we added 25 µM pantothenate which resulted in up to 95 mg/L n-butanol production (Fig. 5, Additional file 1: Figure S9). These results show that increasing the cytosolic acetyl-CoA synthesis by adhE^A267T/E568K^ does improve n-butanol production, especially when combined with elevated CoA synthesis. It should be noted that although we used butyraldehyde dehydrogenase eutE in pVS6, which was also shown to convert acetaldehyde into acetyl-CoA [22], we could not observe a clear improvement in n-butanol production due to this activity.

## Disruption of glycerol synthesis via chromosomal integration of the n-butanol pathway genes into *GPD2* further increases n-butanol production

*GPD2* encodes glycerol-3-phosphate dehydrogenase II which is the major enzyme for glycerol production in yeast under anaerobic conditions [35]. Glycerol production is used to re-oxidize a surplus of NADH. Deletion of *GPD2* would further increase NADH levels in the *adh* deletion mutants [36] and could therefore push n-butanol production. Moreover, in order to overcome the negative effects of using different plasmids we decided to integrate *coaA* into the *ADH6* locus of VSY8 (resulting in strain VSY9), and the n-butanol pathway genes of pVS6 into the *GPD2* locus of strain VSY9, finally resulting in strain VSY10. Fermentations of strain VSY10 resulted in n-butanol titers up to 108 mg/L, which is significantly higher compared to its plasmid-based parental strain VSY8 (pVS6, pVS5_4; 75 mg/L) (Fig. 5). The increase might be due to both the stable integration of n-butanol pathway genes into the genome as well as reduced glycerol production [calculated yield for glycerol: 0.17 g/g glucose in VSY10 and 0.27 g/g in VSY8 (pVS6, pVS5_4)] (Additional file 1: Figure S6). Nevertheless, VSY10 did not consume all of the glucose (Additional file 1: Figure S5). This might be due to deletions of *adh1*-*6*, *sfa1*, and *gpd2*, resulting in inefficient NADH oxidation. The capacity of the introduced n-butanol pathway is obviously not strong enough to compensate this deficiency. This can also be seen by measured OD_600_ values. Without deletions, the wildtype is able to reach an OD_600_ of about 6, but all deletion strains reached not more than an OD_600_ of about 2, except for VSY8 (pVS6), that produced ethanol instead of n-butanol (Additional file 1: Figure S7). As a result of incomplete glucose consumption the n-butanol yield of VSY10 (0.01 g/g glucose) is much higher than for all fermentations with VSY8 (up to 0.0057 g/g) (Additional file 1: Figure S4). The addition of 25 µM pantothenate increased the n-butanol yield of strain VSY10 up to 0.012 g/g (Additional file 1: Figure S4), resulting in a titer of 130 ± 20 mg/L (Fig. 5, Additional file 1: Figure S10). This is to our knowledge the highest titer reported so far for n-butanol production in synthetic media in yeast via an acetoacetyl-CoA-derived n-butanol pathway.

## Conclusions

Compared to bacterial systems, n-butanol production in *S. cerevisiae* via heterologous acetoacetyl-CoA-derived pathways is very low. The reason for this is not fully understood. Our results confirm that several reasons contribute to the low efficiency of n-butanol production in yeast. By stepwise engineering various aspects of n-butanol production, we could gradually increase n-butanol titers and yields. Of course, a very important aspect was the composition of the heterologous pathway. By testing various isoenzymes for the different reactions, we could establish a functional pathway. n-Butanol production was then increased by stable expression of the heterologous genes. Expression from centromeric plasmids was clearly superior to expression from multicopy plasmids (Table 2). Other important aspects were the elevation of acetaldehyde levels and NADH by reduction of alcohol dehydrogenase activities and glycerol synthesis. Moreover, increasing acetyl-CoA availability by means of a mutant form of an acetylating acetaldehyde dehydrogenase from *E. coli* further improved n-butanol production. However, our results indicate that obviously free CoA levels in *S. cerevisiae* are limiting n-butanol production. By increasing synthesis of CoA via overexpression of pantothenate kinase coaA of *E. coli* and by feeding pantothenate to the medium, n-butanol production levels could be increased up to 130 ± 20 mg/L, the highest level ever reached in yeast via an acetoacetyl-CoA-derived pathway in synthetic medium.

## Methods

### Strains and media

All yeast strains used in this work are listed in Table 1. The *adh* deletion strains are based on strain JDY4 (*MATa; ura3*-*52; trp1*-*289; leu2*-*3_112; his3* Δ*1; MAL2*-*8C; SUC2 adh1::loxP adh3::loxP adh5::kanMX*) (lab stocks Boles group), which was constructed from strain CEN.PK484 (EUROSCARF, Frankfurt).

*S. cerevisiae* was cultured in YEPD medium (10 g/L yeast extract, 20 g/L bacteriological peptone, 20 g/L glucose) or synthetic minimal (SMD) medium (1.7 g/L yeast nitrogen base without amino acids, 5 g/L ammonium sulfate, 20 mM monopotassium phosphate, supplemented with uracil (0.171 mM), leucine (0.439 mM), tryptophan (0.093 mM), and histidine (0.124 mM), 20 g/L glucose) at 30°. Synthetic media were adjusted to pH 6.3 with potassium hydroxide. The carbon source was autoclaved separately and added afterwards. If needed, the medium was supplemented with G418 (200 μg/mL), hygromycin B (200 μg/mL), and/or clonNAT/nourseothricin (100 μg/mL) for selection of *kanMX*, *hphNT1*, and/or *natNT2* markers, respectively.

*Escherichia coli* DH10b was used for cloning procedures and was grown in lysogeny broth (LB) at 37 °C with 100 μg/ml ampicillin for plasmid selection.

### Plasmid and strain construction

Plasmids were assembled via homologous recombination in yeast with up to 20 overlapping fragments with 50 bp overlaps. All heterologous genes were codon optimized according to the yeast glycolytic codon usage [37]. The mitochondrial targeting sequence of ^Eg^*ter* was excluded [38] and the start codon of ^Ca^*bdhB* and ^Sco^*ccr* was changed from GTG into ATG. In Additional file 2: Table S2, a list of used genes with identifiers is available. Restriction sites were included in order to change sequences. If sequences within a plasmid were changed, the assembly of the corresponding backbone and the new sequence was performed via Gibson Assembly method [39]. Yeast was transformed according to protocols by Gietz and Schiestl [40, 41] with single fragments generated by PCR using primers shown in Additional file 2: Table S3. The assembled plasmids were recovered by yeast DNA preparations and transformed in *E. coli* for amplification by standard procedures. Restriction digestion analysis and DNA sequencing verified the constructs. The integrative plasmids were generated by successive excision of the 2 μ or CEN6ARS4 origin and the AmpColE1 region and the cassette was integrated via homologous recombination with 400 bp overhangs. Marker rescue was carried out by the loxP/cre recombinase system with *kanMX*, *hphNT1*, or *natNT2* markers. Genome integrations and deletions were confirmed by PCR analysis; primers are listed in Additional file 2: Table S3.

### Fermentations and n-butanol production analysis

To test the n-butanol production of the different strains, pre-cultures were inoculated in YEPD media and grown aerobically to exponential phase. After washing steps, 40 mL SMD medium was inoculated with a starting OD_600_ of 0.3 and grown for at least 100 h. These batch fermentations were carried out semi-anaerobically with a rubber plug and a fermentation lock-sealed 100-mL flasks on a magnetic stirrer with 120 rpm at 30°. Samples were collected at 0, 6, 25, 50, 74, and 100 h for determination of cell density and for HPLC analysis through an inserted, sterile needle and syringe.

To quantify n-butanol concentrations, the samples were centrifuged and 450 µl of the supernatant was mixed with 50 µl 50 % (w/v) 5-sulfosalicylic acid and centrifuged again (10 min, 4 °C, 16,000×*g*). The samples were analyzed in a UHPLC + system by Thermo Scientific (Dionex UltiMate 3000) equipped with a HyperREZ XP Carbohydrate H + 8 μm column and a refractive index detector (Thermo Shodex RI-101). The HPLC was operated at 65 °C with 5 mM sulfuric acid and a flow rate of 0.6 ml/min. n-Butanol, glucose, ethanol, glycerol, and pyruvate were used as standards with concentrations of 0.5 to 20 g/L. Additional standard concentrations for n-butanol were from 0.0125 up to 0.25 g/L.

### Enzyme assays

For enzyme assays, CEN.PK113-5D with the respective plasmid was inoculated in 50 ml YEPD liquid medium with corresponding antibiotics and grown aerobically to an OD_600_ of 0.8–1. The cells were centrifuged and washed with water. The cell pellet was resuspended in 500 µl of the respective enzyme buffer and then lysed by the addition of 2/3 volumes glass beads (∅ 0.25–0.5 mm) and 10 min of shaking on a VXR basic Vibrax (IKA) at 2000 rpm. Cell debris was pelleted by centrifugation (10 min, 16,000×*g*, 4 °C) and the supernatant was used as a cell extract. Protein concentrations were measured by the Bradford method with a Roti-Quant Kit (Carl-Roth GmbH and Co). All enzyme assays were performed using the Ultrospec 2100 pro spectrophotometer (GE Healthcare, USA) at 30 °C under aerobic conditions. For each reaction, a volume of 200 µl was used, whereas 10 µl of cell extract with protein concentrations of 0.05–5 µg/µl was used. The determination of enzyme activity was carried out by calculating the slope of linear sections of absorbance versus time, using GE Healthcare\Datrys\Resolution and the respective molar extinction coefficients.

Thiolase (Erg10, thlA) activity was measured with 0.2 mM acetoacetyl-CoA and 0.2 mM CoA as substrates and the decrease in acetoacetyl-CoA concentration was measured at 303 nm. The reaction buffer contained 100 mM Tris–HCl, 10 mM MgCl_2_, and 1 mM DTT with pH 8. The reaction was started by the addition of cell extract. For determination of enzyme activity, the molar extinction factor ε = 14 mM^−1^cm^−1^ was used (based on [5, 15]).

Hydroxybutyryl-CoA dehydrogenase (hbd) activity was measured with 0.1 mM acetoacetyl-CoA as a substrate and 0.23 mM NADH as a cofactor. The decrease in NADH concentration at 340 nm was monitored. The reaction buffer contained 50 mM MOPS, 15 mM MgCl_2_, 1 mM EDTA, and 5 mM DTT with pH 7.3. The reaction was started by the addition of acetoacetyl-CoA [42].

Crotonase (crt) activity was measured with 0.1 mM crotonyl-CoA as a substrate. The decrease of absorption at 263 nm (disruption of the α-β unsaturation of crotonyl-CoA) was monitored. The reaction buffer contained 100 mM Tris–HCl, pH 7.6. The reaction was started by the addition of cell extract. For determination of enzyme activity, the molar extinction factor ε = 6.7 mM^−1^cm^−1^ was used [7].

Trans-2-enoyl-CoA reductase (ter) activity was measured with 0.5 mM crotonyl-CoA as a substrate and 0.46 mM NADH as a cofactor and 2 µM FAD. The decrease in NADH concentration at 340 nm was monitored. The reaction buffer contained 100 mM potassium phosphate, pH 6.2. The reaction was started by the addition of crotonyl-CoA [38].

Butyraldehyde dehydrogenase (adhE2, eutE) activity was measured with 1 mM butyryl-CoA as a substrate and 300 µM NADH as a cofactor. The decrease in NADH concentration at 340 nm was monitored. The reaction buffer contained 100 mM Tris–HCl and 5 mM DTT at pH 7.5. The reaction was started by the addition of butyryl-CoA [7].

Butanol dehydrogenase (adhE2, bdhB) activity was measured with 50 mM butyraldehyde as a substrate and 300 µM NADH as a cofactor. The decrease in NADH concentration at 340 nm was monitored. The reaction buffer contained 100 mM Tris–HCl and 5 mM DTT at pH 7.5. The reaction was started by the addition of cooled butyraldehyde [7].

Acetyl-CoA:malonyl-CoA acyltransferase (nphT7) activity was measured with 0.4 mM acetyl-CoA and 0.4 mM malonyl-CoA as substrates and the increase in acetoacetyl-CoA concentration was measured at 303 nm. The reaction buffer contained 100 mM Tris–HCl, 10 mM MgCl_2_, and 1 mM DTT with pH 8. The reaction was started by the addition of cell extract. For determination of enzyme activity, the molar extinction factor ε = 14 mM^−1^cm^−1^ was used (based on [5, 28]).

Crotonyl-CoA reductase (ccr) activity was measured with 0.5 mM crotonyl-CoA as a substrate and 0.46 mM NADPH as a cofactor. The decrease in NADPH concentration at 340 nm was monitored. The reaction buffer contained 100 mM potassium phosphate, pH 6.2. The reaction was started by the addition of crotonyl-CoA (based on [38]).

## Additional files

10.1186/s13068-016-0456-7 n-Butanol production with different variants of acetoacetyl-CoA derived n-butanol pathways in *S. cerevisiae* strain CEN.PK113-5D.** Figure S2.** Comparison of n-butanol production with and without overexpression of *coaA* (pVS5_4) and *adhE* ^A267T/E568K^ (pVS4) in VSY0 (Δ*adh1-5*).** Figure S3.** n-Butanol production with n-butanol pathway (pVS6) and *FEN2* overexpression under control of the *MET25* promoter (pVS5_6) in CEN.PK113-5D with addition of methionine.** Figure S4.** Comparison of n-butanol yields of different strains.** Figure S5.** Consumption of glucose in semi-anaerobic fermentations started with 20 g/L glucose.** Figure S6.** Ethanol and glycerol production in semi-anaerobic fermentations.** Figure S7.** OD_600_ after 74 h in semi-anaerobic fermentations.** Figure S8.** n-Butanol production with n-butanol pathway (pVS6) and *coaA* overexpression (pVS5_4) in VSY0 (Δ*adh1-5*) with addition of pantothenate.** Figure S9.** n-Butanol production with n-butanol pathway (pVS6) and *coaA* overexpression (pVS5_4) in VSY8 (Δ*adh1-5 sfa1*, integrated *adhE* ^A267T/E568K^) with addition of pantothenate.** Figure S10.** n-Butanol production of strain VSY10 (Δ*adh1-5 sfa1 adh6 gpd2* with integrated *adhE* ^A267T/E568K^, *coaA* and n-butanol pathway) with addition of pantothenate.
10.1186/s13068-016-0456-7 Yeast promoters and terminators used for the expression of n-butanol pathway genes in this study. **Table S2.** Genes used in this study and their corresponding identifiers. **Table S3.** Relevant primers for this study. **Table S4.** Statistical analysis of n-butanol production.

## Abbreviations

OD_600_: optical density at 600 nm
SMD: synthetic minimal medium containing glucose
ADH: alcohol dehydrogenase
