# Supplementary material for: n-Butanol production in Saccharomyces cerevisiae is limited by the availability of coenzyme A and cytosolic acetyl-CoA
Source: Biotechnol Biofuels. 2016 Feb 24;9:44. doi: 10.1186/s13068-016-0456-7 (PMC4765181; doi:10.1186/s13068-016-0456-7)
Supplement: Supplementary file 1 — 10.1186/s13068-016-0456-7 n-Butanol production with different variants of acetoacetyl-CoA derived n-butanol pathways in S. cerevisiae strain CEN.PK113-5D. Figure S2. Comparison of n-butanol production with and without overexpression of coaA (pVS5_4) and adhE A267T/E568K (pVS4) in VSY0 (Δadh1-5). Figure S3. n-Butanol production with n-butanol pathway (pVS6) and FEN2 overexpression under control of the MET25 promoter (pVS5_6) in CEN.PK113-5D with addition of methionine. Figure S4. Comparison of n-butanol yields of different strains. Figure S5. Consumption of glucose in semi-anaerobic fermentations started with 20 g/L glucose. Figure S6. Ethanol and glycerol production in semi-anaerobic fermentations. Figure S7. OD600 after 74 h in semi-anaerobic fermentations. Figure S8. n-Butanol production with n-butanol pathway (pVS6) and coaA overexpression (pVS5_4) in VSY0 (Δadh1-5) with addition of pantothenate. Figure S9. n-Butanol production with n-butanol pathway (pVS6) and coaA overexpression (pVS5_4) in VSY8 (Δadh1-5 sfa1, integrated adhE A267T/E568K) with addition of pantothenate. Figure S10. n-Butanol production of strain VSY10 (Δadh1-5 sfa1 adh6 gpd2 with integrated adhE A267T/E568K, coaA and n-butanol pathway) with addition of pantothenate. [file 13068_2016_456_MOESM1_ESM.pdf]

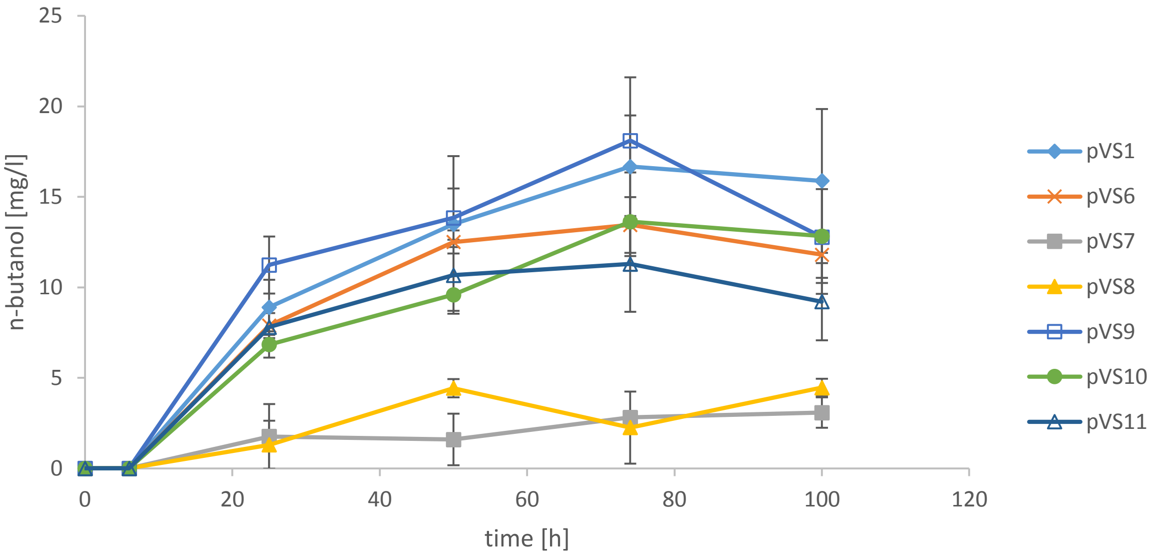

Fig. S1: n-Butanol production with different variants of acetoacetyl-CoA derived n-butanol pathways in *S. cerevisiae* strain CEN.PK113-5D. The n-butanol concentrations of semi-anaerobic fermentations in SMD medium within 100 h are shown. Six different variants are compared: pVS1 (diamond), pVS6 (cross), pVS7 (square), pVS8 (triangle), pVS9 (open square), pVS10 (circle), pVS11 (open triangle). Error bars represent the standard deviation of three independent replicates.

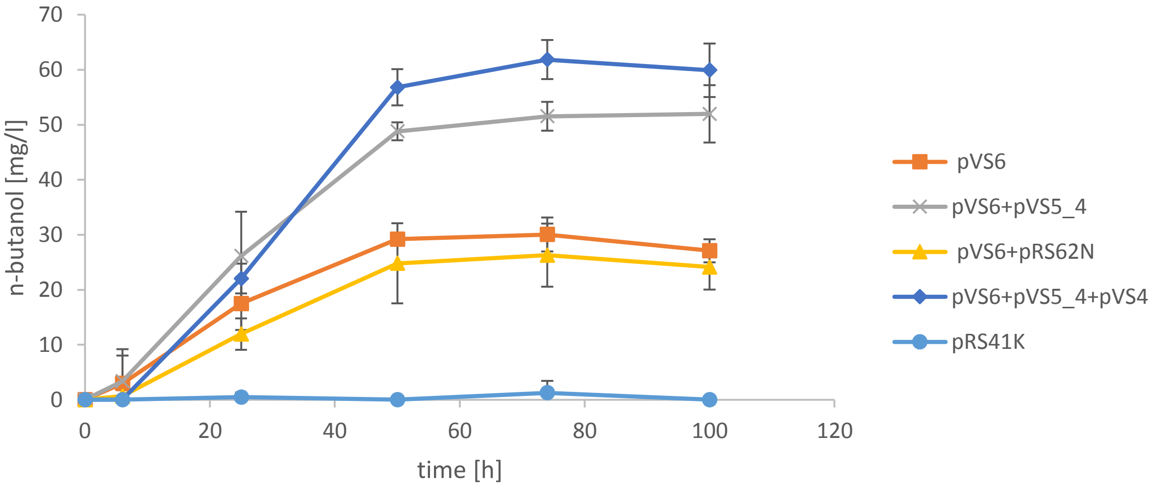

Fig. S2: Comparison of n-butanol production with and without overexpression of *coaA* (pVS5_4) and *adhE*$^{A267T/E568K}$ (pVS4) in VSY0 (Δ*adh1-5*). Different combinations of plasmids were used for fermentations in SMD medium: pVS6 (square), pVS6+pVS5_4 (cross), as a control pVS6 with an empty vector instead of pVS5_4 (triangle), pVS6+pVS5_4+pVS4 (diamond) and an empty vector (circle). Error bars represent the standard deviation of three independent replicates.

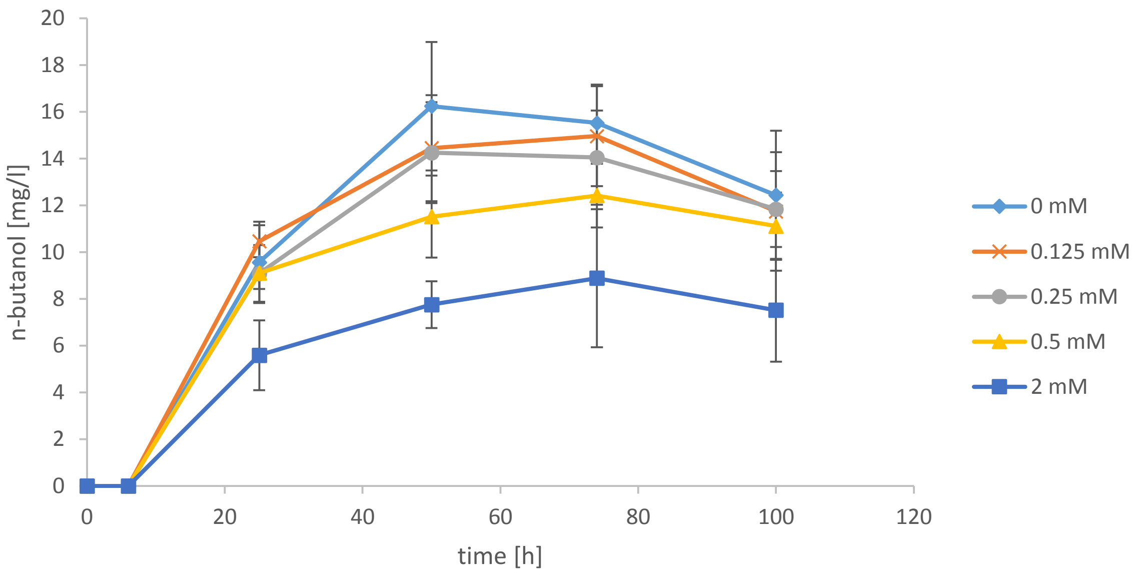

Fig. S3: n-Butanol production with n-butanol pathway (pVS6) and *FEN2* overexpression behind the *MET25* promoter (pVS5_6) in CEN.PK113-5D with addition of methionine. Five different concentrations of methionine were added to SMD medium: 0 mM (diamond), 0.125 mM (cross), 0.25 mM (circle), 0.5 mM (triangle), 2 mM (square). Error bars represent the standard deviation of three independent replicates.

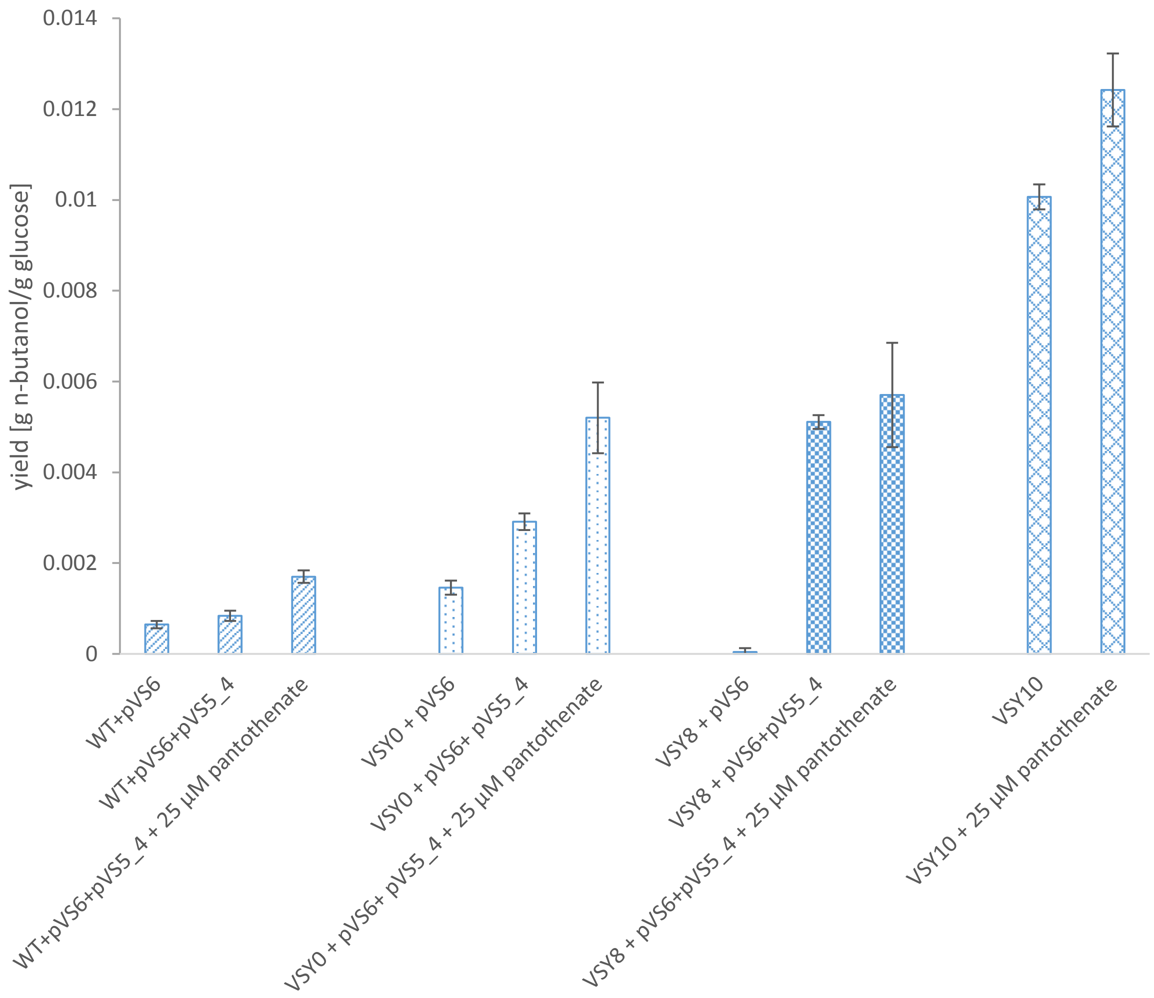

Fig. S4: Comparison of n-butanol yields of different strains. The amount of n-butanol after 74 h is calculated per g consumed glucose after 74 h of semi-anaerobic fermentations in SMD medium. Compared are fermentations of the wildtype CEN.PK113-5D, VSY0 (Δ*adh1-5*), and VSY8 (Δ*adh1-5 sfa1* with *adhE*$^{A267T/E568K}$) with plasmid pVS6 (n-butanol pathway), with or without *coaA* overexpression (pVS5_4), and strain VSY10 (Δ*adh1-6 sfa1 gpd2,* with n-butanol pathway genes of pVS6, *coaA* and *adhE*$^{A267T/E568K}$), in the absence or presence of additional 25 µM pantothenate, in SMD medium. Error bars represent the standard deviation of three independent replicates and a statistical analysis is shown in Tab. S4.

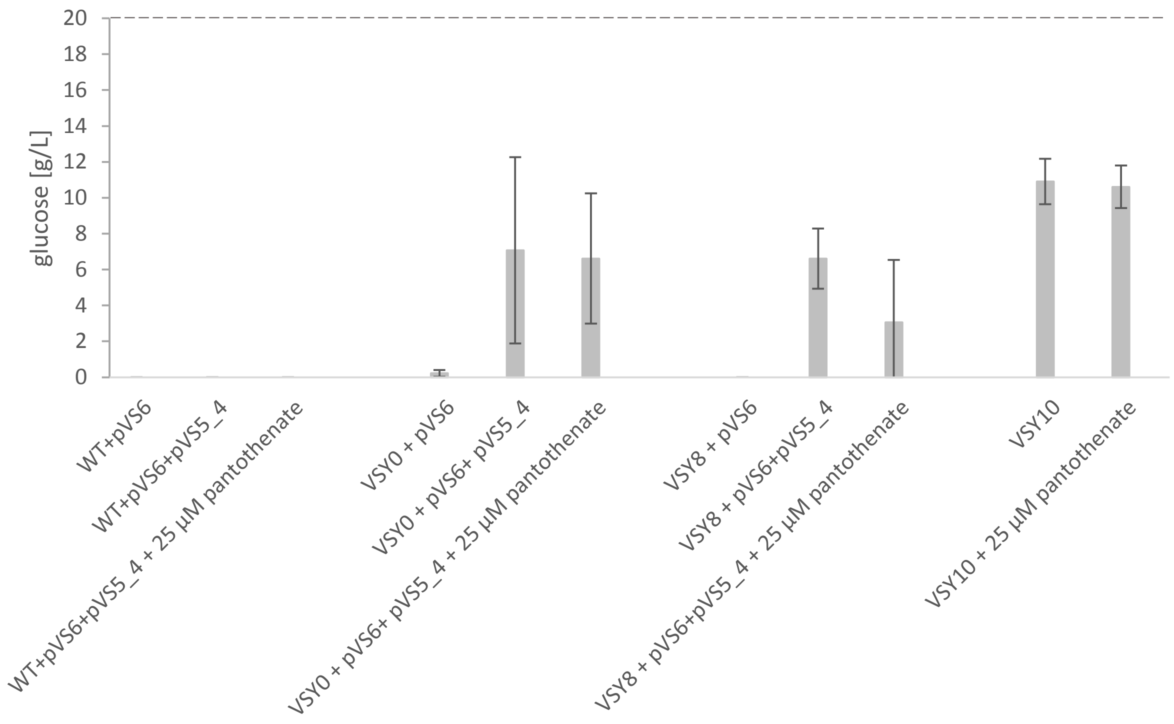

Fig. S5: Consumption of glucose in semi-anaerobic fermentations started with 20 g/L glucose. Residual glucose after 74 h fermentations in SMD medium is shown. Compared are fermentations of the wildtype CEN.PK113-5D, VSY0 (Δ*adh1-5*), and VSY8 (Δ*adh1-5 sfa1* with *adhE*$^{A267T/E568K}$) with plasmid pVS6 (n-butanol pathway), with or without *coaA* overexpression (pVS5_4), and strain VSY10 (Δ*adh1-6 sfa1 gpd2,* with n-butanol pathway genes of pVS6, *coaA* and *adhE*$^{A267T/E568K}$), in the absence or presence of additional 25 µM pantothenate, in SMD medium. Error bars represent the standard deviation of three independent replicates.

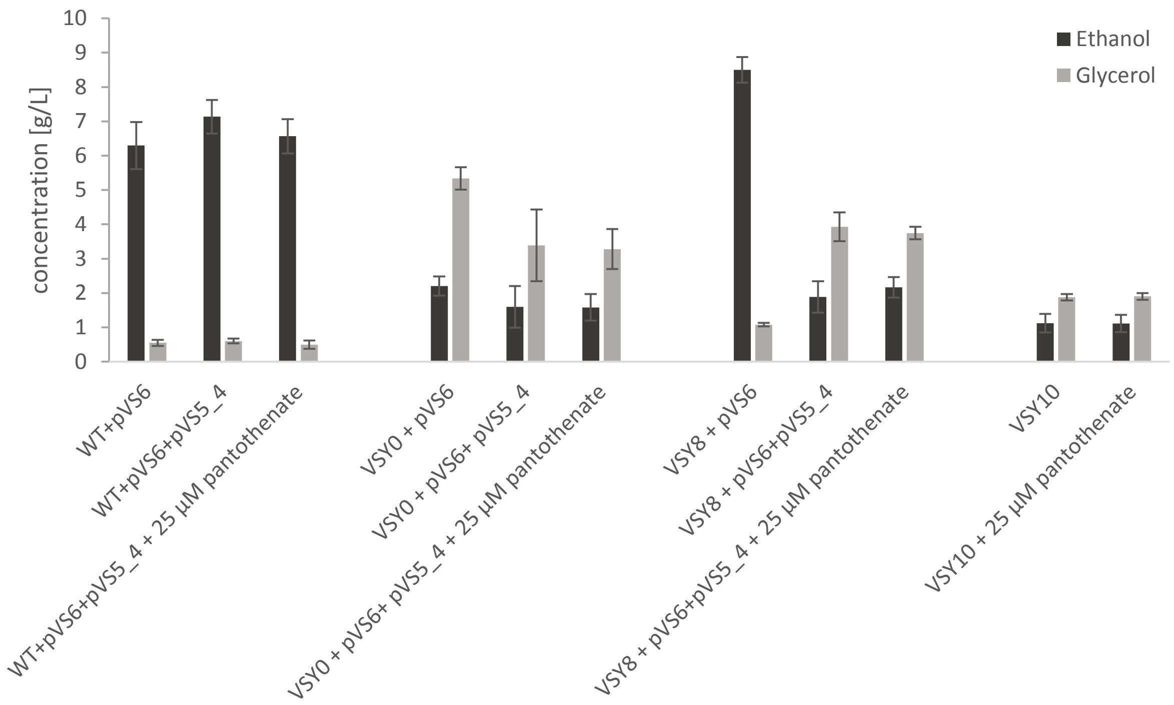

Fig. S6: Ethanol and glycerol production in semi-anaerobic fermentations. The amounts of ethanol and glycerol after 74 h fermentations in SMD medium are shown. Compared are fermentations of the wildtype CEN.PK113-5D, VSY0 (Δ*adh1-5*), and VSY8 (Δ*adh1-5 sfa1* with *adhE*$^{A267T/E568K}$) with plasmid pVS6 (n-butanol pathway), with or without *coaA* overexpression (pVS5_4), and strain VSY10 (Δ*adh1-6 sfa1 gpd2*, with n-butanol pathway genes of pVS6, *coaA* and *adhE*$^{A267T/E568K}$), in the absence or presence of additional 25 µM pantothenate, in SMD medium. Error bars represent the standard deviation of three independent replicates.

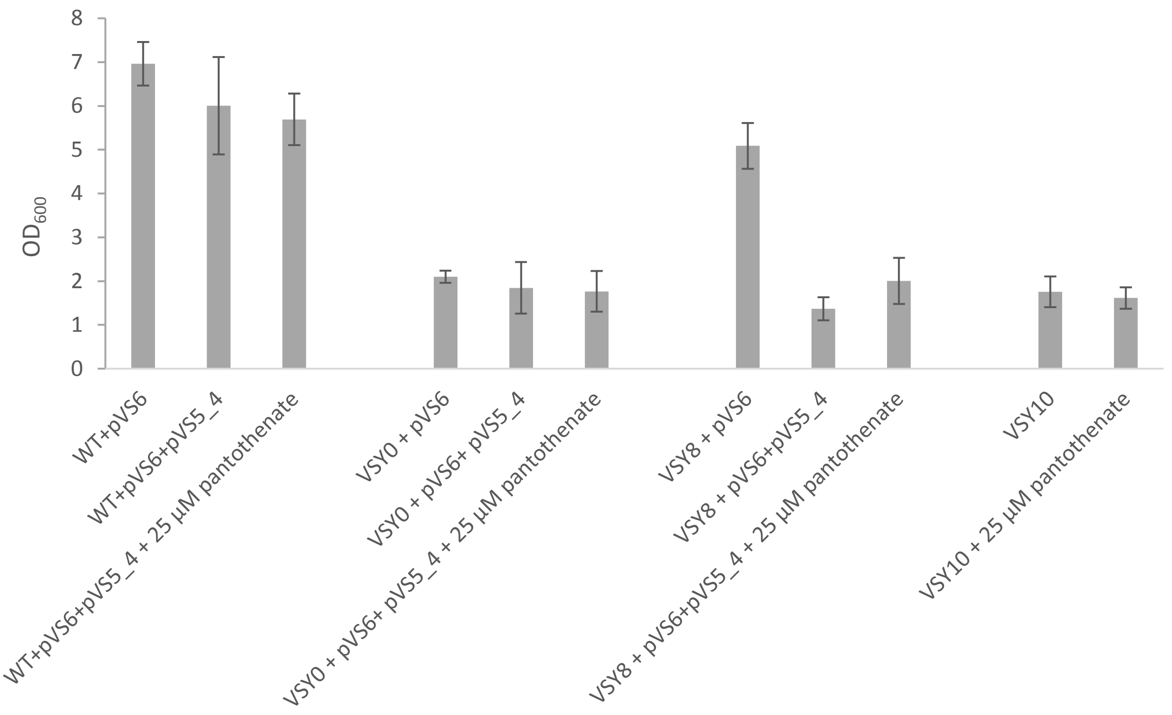

Fig. S7: OD_{600} after 74 h in semi-anaerobic fermentations. The OD_{600} after 74 h fermentations, that were started with an OD_{600} of 0.3 in SMD medium are shown. Compared are fermentations of the wildtype CEN.PK113-5D, VSY0 (Δ*adh1-5*), and VSY8 (Δ*adh1-5 sfa1* with *adhE*$^{A267T/E568K}$) with plasmid pVS6 (n-butanol pathway), with or without *coaA* overexpression (pVS5_4), and strain VSY10 (Δ*adh1-6 sfa1 gpd2*, with n-butanol pathway genes of pVS6, *coaA* and *adhE*$^{A267T/E568K}$), in the absence or presence of additional 25 µM pantothenate, in SMD medium. Error bars represent the standard deviation of three independent replicates.

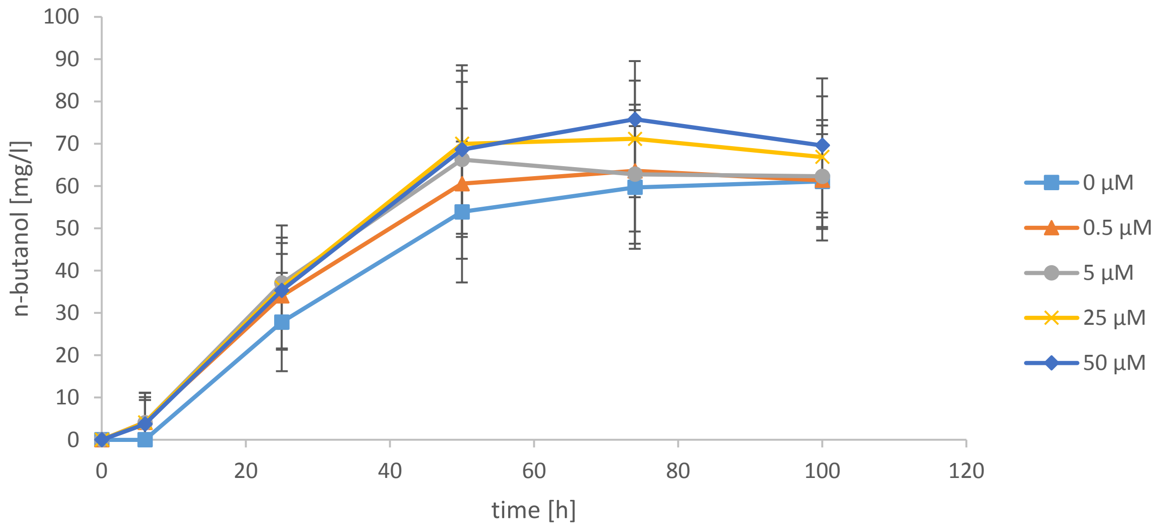

Fig. S8: **n-Butanol production with n-butanol pathway (pVS6) and *coaA* overexpression (pVS5_4) in VSY0 (Δ*adh1-5*) with addition of pantothenate.** Five different concentrations of pantothenate were added to SMD medium: 0 µM (square), 0.5 µM (triangle), 5 µM (circle), 25 µM (cross), 50 µM (diamond). Error bars represent the standard deviation of three independent replicates.

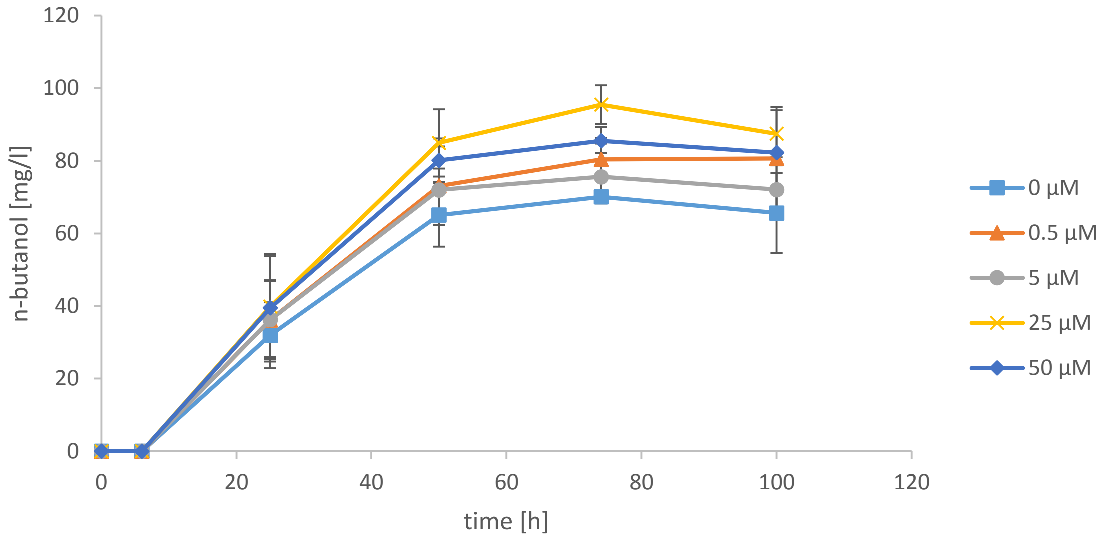

Fig. S9: n-Butanol production with n-butanol pathway (pVS6) and *coaA* overexpression (pVS5_4) in VSY8 (*Δadh1-5 sfa1*, integrated *adhE*$^{A267T/E568K}$) with addition of pantothenate. Five different concentrations of pantothenate were added to SMD medium: 0 µM (square), 0.5 µM (triangle), 5 µM (circle), 25 µM (cross), 50 µM (diamond). Error bars represent the standard deviation of three independent replicates.

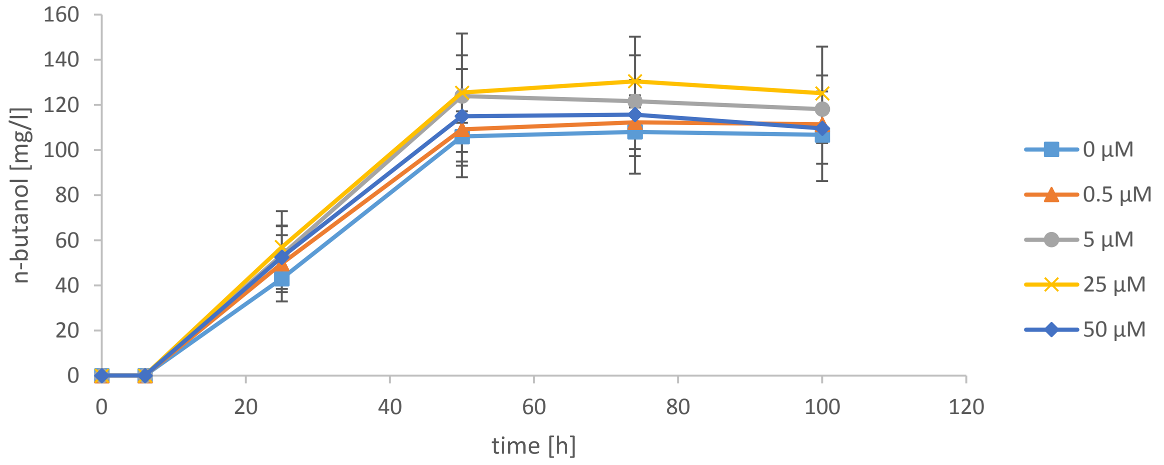

Fig. S10: n-Butanol production of strain VSY10 (*Δadh1-5 sfa1 adh6 gpd2* with integrated *adhE*$^{A267T/E568K}$, *coaA* and n-butanol pathway) with addition of pantothenate. Five different concentrations of pantothenate were added to SMD medium: 0 µM (square), 0.5 µM (triangle), 5 µM (circle), 25 µM (cross), 50 µM (diamond). Error bars represent the standard deviation of three independent replicates.
