# Supplementary material for: n-Butanol production in Saccharomyces cerevisiae is limited by the availability of coenzyme A and cytosolic acetyl-CoA
Source: Biotechnol Biofuels. 2016 Feb 24;9:44. doi: 10.1186/s13068-016-0456-7 (PMC4765181; doi:10.1186/s13068-016-0456-7)
Supplement: Supplementary file 2 — 10.1186/s13068-016-0456-7 Yeast promoters and terminators used for the expression of n-butanol pathway genes in this study. Table S2. Genes used in this study and their corresponding identifiers. Table S3. Relevant primers for this study. Table S4. Statistical analysis of n-butanol production. [file 13068_2016_456_MOESM2_ESM.pdf]

Table S1: Yeast promoters and terminators used for the expression of n-butanol pathway genes in this study. DNA sequences were amplified from chromosomal DNA of CEN.PK2-1C. When not otherwise indicated, promoters were taken from 1-500 bp before and terminators 1-300 bp behind the respective open reading frames.

promoter	gene	terminator
pHXT7*	^{Sc}*ERG10,* ^{Ca}*thlA,* ^{Ss}*nphT7*	tVMA16
pPGK1	^{Ca}*hbd*	tEFM1
pTPI1	^{Ca}*crt*	tYHI9
pPYK1	^{Eg}*ter,* ^{Td}*ter,* ^{Sco}*ccr*	tIDP1
pADH1**	^{Ca}*adhE2,* ^{Ca}*bdhB*	tRPL3
pTDH3	^{Ec}*eutE*	tRPL41B
pPFK1	^{Ec}*adhE* $^{A267T/E568K}$	tDIT1
pFBA1, pMET25	^{Sc}*FEN2*	tPRC1
pPMA1***, pMET25	^{Ec}*coaA*	tVMA2
pGPM1****	^{Sc}*CAB2*	tNAT5

* (-1 to -389 bp; [1])
** (-1 to -800 bp; [2])
*** (-1 to-575 bp, -700 to -925 bp; [3])
**** (-1 to -395 bp; [4])

Table S2: Genes used in this study and their corresponding identifiers.
Genes from *Saccharomyces cerevisiae* (Sc), *Clostridium acetobutylicum* (Ca), *E.coli* (Ec), *Euglena gracilis* (Eg), *Streptomyces collinus* (Sco), *Streptomyces sp*. CL190 (Ss), *Treponema denticola* (Td) are indicated by prefixes in superscript.

gene	identifier
^{Sc}*ERG10*	YPL028W
^{Ca}*hbd*	CA_C2708
^{Ca}*crt*	CA_C2712
^{Eg}*ter*	Q5EY90
^{Td}*ter*	TDE_0597
^{Ca}*adhE2*	CA_P0035
^{Ec}*eutE*	G7285
^{Ca}*thlA*	CA_C2873
^{Ca}*bdhB*	CA_C3298
^{Ss}*nphT7*	D7URV0
^{Sco}*ccr*	AAA92890.1
^{Ec}*adhE*	EG10031
^{Sc}*FEN2*	YCR028C
^{Ec}*coaA*	EG10922
^{Sc}*CAB2*	YIL083C

Table S3: Relevant primers for this study

The abbreviations within the primer names were used as follows: forward primer (fw), reverse primer (rev), overlap (ov).

Primer name	Sequence 5‘-3‘	Description
vsp70_2µ_ovInt3'GPD2_fw	TGACGGCTCTCCAGTCAGGCGCCGTCGACACGCATTT AAGCATAAACACG	construction pVS1
vsp71_2µ_ovAmp_rev	CCCCGAAAAGTGCCACCTGAGGCCTGTCGACAACGA AGCATCTGTGCTTC	construction pVS1
vsp72_AmpColE1_ov2µ_fw	GAAGCACAGATGCTTCGTTGTCGACAGGCCTCAGGT GGCACTTTTCGGGG	construction pVS1
vsp73_AmpColE1_ovInt5'GPD2_rev	GGTAATTCCTTGCTCCAGTTTTCCTGCAGGAGGCCTC GCGTTGCTGGCGTTTTTCC	construction pVS1
vsp74_Int5'GPD2_ovAmpColE1_fw	ACGCCAGCAACGCGAGGCCTCCTGCAGGAAAACTGG AGCAAGGAATTACC	construction pVS1
vsp75_Int5'GPD2_ovtVMA16_rev	AAAATTACTACGCCCTCAATATGCTAGCCCTGCAGGA AAAAAAACTAGAAAAAAAGTAACAAG	construction pVS1
vsp76_tVMA16_ovInt5'GPD2_fw	TTCTAGTTTTTTTTCCTGCAGGGCTAGCATATTGAGGG CGTAGTAATTTTAAAGAAGAAC	construction pVS1
vsp77_tVMA16_ovERG10_rev	CCTCTATTGTCATTGAAAAGATTTAAGCGCTCAAACC AGGCTTTTC	construction pVS1
vsp78_ERG10_ovtVMA16_fw	AAAAGAAAAGCCTGGTTTGAGCGCTTAAATCTTTTCA ATGACAATAGAGG	construction pVS1
vsp79_ERG10_ovpHXT7_rev	CAAAAAGTTTTTTTAATTTTAATCAAAAAATGTCTCAA AACGTTTACATTG	construction pVS1
vsp80_pHXT7_ovERG10_fw	AATGTAAACGTTTTGAGACATTTTTTGATTAAAATTAA AAAAACTTTTTG	construction pVS1
vsp81_pHXT7_ovpPGK1_rev	GTTTTGTTCTTTTTGCAAACACCCGGGGCTAGCCGTA GGAACAATTTCGGGC	construction pVS1
vsp82_pPGK1_ovpHXT7_fw	CCGAAATTGTTCCTACGGCTAGCCCCGGGTGTTTGCA AAAAGAACAAAAC	construction pVS1
vsp83_pPGK1_ovHbd_rev	CCAATAACACAAACCTTCTTCATTGTTTTATATTTGTTG TAAAAAGTAG	construction pVS1
vsp84_Hbd_ovpPGK1_fw	TCTACTTTTTACAACAAATATAAAACAATGAAGAAGG TTTGTGTTATTGG	construction pVS1
vsp85_Hbd_ovtEFM1_rev	GAATTTTATACTTAGGCTACAGATCAAAGTTACTTAG AGTAATCGTAGAAACCC	construction pVS1
vsp86_tEFM1_ovHbd_fw	TTCTACGATTACTCTAAGTAACTTTGATCTGTAGCCTA AGTATAAAATTC	construction pVS1
vsp87_tEFM1_ovYHI9_rev	GAACTTTTCGATACAAAAAGCGGCCGCCCCGGGTACT GAAATTCATGACACAAATAACG	construction pVS1
vsp88_tYHI9_ovEFM1_fw	TGTCATGAATTTCAGTACCCGGGGCGGCCGCTTTTTG TATCGAAAAGTTCATCAG	construction pVS1
vsp89_tYHI9_ovCrt_rev	GATTGAAGGTTTCAAGAACAGATAAGATTCTAAACGC ATAGTTGTAAGG	construction pVS1
vsp90_Crt_ovtYHI9_fw	ACCTTACAACTATGCGTTTAGAATCTTATCTGTTCTTG AAACCTTCAATC	construction pVS1
vsp91_Crt_ovpTPI1_rev	CTACAAAAAACACATACATAAACTAAAAATGGAATTG AACAACGTCATC	construction pVS1
vsp92_pTPI1_ovCrt_fw	AGATGACGTTGTTCAATTCCATTTTTAGTTTATGTATG TGTTTTTTGTAG	construction pVS1
vsp93_pTPI1_ovloxP_rev	TTATATTAAGGGTTGTCGCCTAGGGCGGCCGCATTTA AACTGTGAGGACCTTAATAC	construction pVS1
vsp94_loxP_ovpTPI1_fw	GGTCCTCACAGTTTAAATGCGGCCGCCCTAGGCGACA ACCCTTAATATAACTTCG	construction pVS1
vsp95_loxP_ovpPYK1_rev	AAAATCTCCCAATCTGTGGCGCGCCCCTAGGGATCTG ATATCACCTAATAACTTCG	construction pVS1
vsp96_pPYK1_ovloxP_fw	TATTAGGTGATATCAGATCCCTAGGGGCGCGCCACA GATTGGGAGATTTTCATAGTAGAC	construction pVS1
vsp97_pPYK1_ovegTer_rev	GGTGGTGGTGAACATAGCCATTGTGATGATGTTTTAT TTGTTTTGATTG	construction pVS1
vsp98_egTer_ovpPYK1_fw	CCAATCAAAACAAATAAAACATCATCACAATGGCTAT GTTCACCACCACC	construction pVS1

vsp99_egTer_ovtIDP1_rev	GTAGATTGGGCTACGTAAATTCGATTATTGTTGAGCAGCAGATGGC	construction pVS1
vsp100_tIDP1_ovegTer_fw	ACTTGCCATCTGCTGCTCAACAATAATCGAATTTACGTAGCCCAATCTAC	construction pVS1
vsp101_tIDP1_ovtRPL3_rev	CAATCTTTGCTACAATGCCGGCGGCGCCAACTTTTGGACGAATTAGAACAGC	construction pVS1
vsp102_tRPL3_ovtIDP1_fw	TTCTAATTCGTCCAAAAGTTGGCGCGCCGCCGGCATTGTAGCAAAGATTGTAAGG	construction pVS1
vsp103tRPL3_ovAdhE2_rev	GAAGGATATCTACATTAAGTCTTTCTAAGAAGTTTTGTTAGAAAATAAATC	construction pVS1
vsp104_AdhE2_ovtRPL3_fw	ATTTATTTTCTAACAAAACTTCTTAGAAAGACTTAATGTAGATATCCTTC	construction pVS1
vsp105_AdhE2_ovpADH1_rev	GCATACAATCAACTATCTCATATACAATGAAGGTTACCAACCAAAAG	construction pVS1
vsp106_pADH1_ovAdhE2_fw	TTCCTTTTGGTTGGTAACCTTCATTGTATATGAGATAGTTGATTGTATGC	construction pVS1
vsp107_pADH1_ovInt3'GPD2_rev	GAGGGGGGGGGAGAGTGTGGCGCCGCCGGCAAGTCCAATGCTAGTAGAGAAGG	construction pVS1
vsp108_Int3'GPD2_ovpADH1_fw	TCTCTACTAGCATTGGACTTGCCGGCGGCGCCACACTCTCCCCCCCCCTC	construction pVS1
vsp109_Int3'GPD2_ov2µ_rev	CGTGTTTATGCTTAAATGCGTGTCGACGGCGCCTGACTGGAGAGCCGTCAGTAGTG	construction pVS1
vsp136_Int3'SFA1_ovpPFK1_fw	CTCATCCTTATATTTTTCACTAGTGGCGCCGAGTACTTAATTAAACTAAGTAAGCATG	construction pVS4
vsp137_Int5'SFA1_ovloxP_rev	GTTATATTAAGGGTTGTCGCCTAGGCCTGCAGGAATTCCATTATTACGTAACTTACAG	construction pVS4
vsp138_loxP_ovInt5'SFA1_fw	TACGTAATAATGGAATTCCTGCAGGCCTAGGCGACAACCCTTAATATAACTTCG	construction pVS4
vsp143_pPFK1_ovInt3'SFA1_rev	CTTAGTTTAATTAAGTACTCGGCGCCACTAGTGAAAAATATAAGGATGAGAAAGTG	construction pVS4
vsp144_pPFK1_ovadhE_fw	TCAATTCAGCGACGTTAGTAACAGCCATCTTTGATATGATTTTGTTTCAG	construction pVS4
vsp145_tDIT1_ovadhE_rev	AGAAGGCTAAGAAGTCCGCTTAATAAAGTAAGAGCGCTACATTGG	construction pVS4
vsp146_adhE_ovtDIT1_fw	GTAGACCAATGTAGCGCTCTTACTTTATTAAGCGGACTTCTTAGCCTTC	construction pVS4
vsp147_adhE_ovpPFK1_rev	CTGAAACAAAATCATATCAAAGATGGCTGTTACTAACGTCGCTG	construction pVS4
vsp150_Leu_fw	AGGTTCCTTGGGTTTGTTGCC	verification of Δadh2
vsp151_Leu_rev	GGCAAAACGACGATCTTCTTAGGG	verification of Δadh2
vsp154_seq1_2µ	GGTAAAATAGCGCTTTCGCG	sequencing pVS1
vsp155_seq2_ColE1	AGGGAGCTTCCAGGGGG	sequencing pVS1
vsp156_seq3_tVMA16	CATACACATGTATCTCAGATATCTC	sequencing pVS1
vsp157_seq4_ERG10	TTTCGTTGTCGAACTTACC	sequencing pVS1
vsp158_seq5_pHXT7	CTTAGAAAAGACAAGTTGAGC	sequencing pVS1
vsp159_seq6_pPGK1	CGTGTGACAACAACAGCC	sequencing pVS1
vsp160_seq7_hbd	TATTGCTATTGGTAAGGATCC	sequencing pVS1
vsp161_seq8_tYHI9	CTCAACTTCTTCTTCGCC	sequencing pVS1
vsp162_seq9_crt	CACCGAAACCTGGGG	sequencing pVS1
vsp163_seq10_pTPI1	TTACAGCAGCCGAGCC	sequencing pVS1
vsp164_seq11_kanMX	GGATTTGCCACTGAGG	sequencing pVS1
vsp165_seq12_kanMX	TCGACATCATCTGCCC	sequencing pVS1
vsp166_seq13_pPYK1	CAGTTATATCATGGTCCCC	sequencing pVS1
vsp167_seq14_egTer	GGTGTCTTGCACAAGGC	sequencing pVS1
vsp168_seq15_egTer	TCTCCGACTTCGCTGG	sequencing pVS1
vsp169_seq16_tRPL3	TCCATTTTGGACTTGGG	sequencing pVS1
vsp170_seq17_adhE2	TGTTAGAAGCGTGAGCC	sequencing pVS1
vsp171_seq18_adhE2	GGTAATCTTGTTAACGTAACCC	sequencing pVS1
vsp172_seq19_adhE2	TGTCAGCGTTAATAGCACC	sequencing pVS1

vsp173_seq20_adhE2	CGTCGTGGTCAATAATACC	sequencing pVS1
vsp174_seq21_pADH1	ATTTTTTTGTCATTGCGG	sequencing pVS1
vsp175_seq22_Int3'GPD2	TCTTTTTCCCCCAACC	sequencing pVS1
vsp181_seq28_hphNT1	AGAGATCTGTTTAGCTTGCC	sequencing pVS4
vsp182_seq29_hphNT1	GTCACGCTTACATTCACG	sequencing pVS4
vsp185_seq32_pPFK1	TGTTGATGACGACAATGG	sequencing pVS4
vsp189_kanMX_ovtADH4_rev	ATAAGGCACACGCATAATTGACGTTTATGAGTTCGTT CGATTTTTTCACTAGTGGATCTGATATCACC	Δadh4 kanMX-deletion cassette construction
vsp190_kanMX_ovpADH4_fw	TAGTTTTTAGTTCGCGCATCACGAGGTACGTGTTTAAT ATGTCAGACGCTGCAGGTCGAC	Δadh4 kanMX-deletion cassette construction
vsp191_LEU2_ovtADH2_rev	ACTTGATAATGAAAACTATAAATCGTAAAGACATAAG AGATCCGCAATCCTCCAATATCAAATTAGG	Δadh2 LEU2- deletion cassette construction
vsp192_LEU2_ovpADH2_fw	AAGCATACAATCAACTATCAACTATTAACTATATCGTA ATACACAAATCAATTGTCCTGTACTTCC	Δadh2 LEU2- deletion cassette construction
vsp193_tADH4_rev	CAAACGTACCGGTCTCGGC	verification Δadh4 and fusion-PCR for deletion cassette
vsp194_pADH4_fw	TGTGTTCAGAAGGATCCCCGG	verification Δadh4 and fusion-PCR for deletion cassette
vsp195_tADH2_rev	GGGTACAACTCACAGGCGAGGG	verification Δadh2 and fusion-PCR for deletion cassette
vsp196_pADH2_fw	GGCAGAGGAGAGCATAGAAATGGGG	verification Δadh2 and fusion-PCR for deletion cassette
vsp197_Int3`SFA1_ov2µ_rev	CGTGTTTATGCTTAAATGCGTGCCGGCGGCGCCTGGA ATTTATGTCACCACC	construction pVS4
vsp198_2µ_ovInt3`SFA1_fw	TGGTGACATAAATTCCAGGCGCCGCCGGCACGCATTT AAGCATAAACACG	construction pVS4
vsp199_2µ_ovAmp_rev	CCCCGAAAAGTGCCACCTGGGCGCGCCGCCGGCAAC GAAGCATCTGTGCTTC	construction pVS4
vsp200_AmpColE1_ov2µ_fw	GAAGCACAGATGCTTCGTTGCCGGCGGCGCGCCCAG GTGGCACTTTTCGGGG	construction pVS4
vsp201_AmpColE1_ovInt5`SFA1_rev	TTAGGAATATATATGAGTGTACCTGCAGGGGCGCGC CCGCGTTGCTGGCGTTTTTCC	construction pVS4
vsp202_Int5'SFA1_ovAmpColE1_fw	AACGCCAGCAACGCGGGCGCGCCCCTGCAGGTACAC TCATATATATTCCTAATTTCC	construction pVS4
vsp205_seq38_adhE	TCTTAGCAGCAGTTTCACC	sequencing pVS4
vsp206_seq39_adhE	CGTGAGTAACAGCGTCC	sequencing pVS4
vsp207_seq40_adhE	AGATGGAGTTACCACCCC	sequencing pVS4
vsp208_seq41_adhE	GATAGCTGGCTTACCGG	sequencing pVS4
vsp209_seq42_pPFK1	AGCTTTCCCAAATAGTGC	sequencing pVS4
vsp210_tADH4_ovkanMX_fw	ATATCAGATCCACTAGTGAAAAAATCGAACGAACTCA TAAACGTC	fusion-PCR for Δadh4 deletion cassette
vsp211_pADH4_ovkanMX_rev	GTCGACCTGCAGCGTCTGACATATTAAACACGTACCT CGTGATGC	fusion-PCR for Δadh4 deletion cassette
vsp212_pADH4_fw	AGCTGTTATGCGCACTACTTGAAAGG	verification Δadh4
vsp213_tADH4_rev	GCATCCAACTGAAACCCACC	verification Δadh4
vsp214_ADH1_fw	CTTCTACGAATCCCACGG	test-PCR within ADH1
vsp215_ADH1_rev	GACCAAACCTCTGGCG	test-PCR within ADH1
vsp216_ADH3_fw	CCACAGCTGCAATCCC	test-PCR within ADH3
vsp217_ADH3_rev	GTGATTTGATCAAACCTCTGC	test-PCR within ADH3
vsp218_ADH5_fw	AGTTCCGGAACCTAAGCC	test-PCR within ADH5
vsp219_ADH5_rev	GATTTGATCAAACCTCTGGC	test-PCR within ADH5
vsp220_ADH4_fw	CAGCTATTGGTCTCTCCGG	test-PCR within ADH4
vsp221_ADH4_rev	TCACCTAATCTCTTCTTGGCC	test-PCR within ADH4
vsp222_ADH2_fw	GGCATGGTGACTGGCC	test-PCR within ADH2
vsp223_ADH2_rev	TGGTATCAGCTCTGTTCCCC	test-PCR within ADH2

vsp235_pADH2_ovLeu2_rev	GTATTACGATATAGTTAATAGTTGATAGTTGATTGTAT GCTTTTTGTAGC	fusion-PCR for Δadh2 deletion cassette
vsp236_tADH2_ovLeu2_fw	GGATCTCTTATGTCTTTACGATTTATAGTTTTCATTATC AAGTATGC	fusion-PCR for Δadh2 deletion cassette
vsp237_tADH2_rev	GAGGGAGACGATTCAGAGGAGC	verification Δadh2
vsp238_pADH2_fw	GGAACACCGGGCATCTCC	verification Δadh2
vsp239_2µ_ovAmp_rev	CCGAAAAGTGCCACCTGAGGCCTGGCGCGCCAACGA AGCATCTGTGCTTC	construction pVS5
vsp240_2µ_ovInt3'ADH6_fw	TAGCGGATGAGGATGTTCCACTAGTGGCGCGCCACG CATTTAAGCATAAACACG	construction pVS5
vsp241_AmpColE1_ovInt5'ADH6_rev	TTTATTATCTATTATATATCACCTGCAGGAGGCCTCGC GTTGCTGGCGTTTTTCC	construction pVS5
vsp242_AmpColE1_ov2µ_fw	AGCACAGATGCTTCGTTGGCGCGCCAGGCCTCAGGT GGCACTTTTCGGGG	construction pVS5
vsp243_Int5'ADH6_ovtPRC1_rev	TACCGAGTTTGTAAGTACAATGTTTAAACCCTGCAGG CTTTCACTTTTCTTTTTCTCC	construction pVS5
vsp244_Int5'ADH6_ovAmp_fw	AACGCCAGCAACGCGAGGCCTCCTGCAGGTGATATA TAATAGATAATAAATCCAGG	construction pVS5
vsp245_tPRC1_ovFEN2_rev	GGCTGAAATTTCTAACCCATTCAGATAAAGCGTGTAT GTGTAGGCATACC	construction pVS5
vsp246_tPRC1_ovInt5'ADH6_fw	AGAAAAGTGAAAGCCTGCAGGGTTTAAACATTGTAC TTACAAACTCGGTAGTTGG	construction pVS5
vsp247_FEN2_ov_pFBA1_rev	CCATAACCAAGTAATACATATTCAAAATGATGAAGGA ATCTAAGTCTATCACTC	construction pVS5
vsp248_FEN2_ov_tPRC1_fw	ACGGTATGCCTACACATACACGCTTTATCTGAATGGG TTAGAAATTTCAG	construction pVS5
vsp249_pFBA1_ovloxP_rev	TTATATTAAGGGTTGTCGCCTAGGGTTTAAACTGGGT CATTACGTAAATAATGATAGG	construction pVS5
vsp250_pFBA1_ovFEN2_fw	GATAGACTTAGATTCCTTCATCATTTTGAATATGTATT ACTTGGTTATGG	construction pVS5
vsp251_loxP_ovpPMA1_rev	GCCTGTTTATGTTTCTCCGTTGCGGCCGCCCTAGGGA TCTGATATCACCTAATAACTTCG	construction pVS5
vsp252_loxP_ovpFBA1_fw	TATTTACGTAATGACCCAGTTTAAACCCTAGGCGACA ACCCTTAATATAACTTCG	construction pVS5
vsp253_pPMA1_ovcoaA_rev	GGTTTGTTCCTTAATAGACATATTGATATTGTTTGATA ATTAAATCTTTC	construction pVS5
vsp254_pPMA1_ovloxP_fw	AGGTGATATCAGATCCCTAGGGCGGCCGCAACGGAG AAACATAAACAGGC	construction pVS5
vsp255_coaA_ovtVMA2_rev	AAAAGCCTTTTTCTTCAGCAACCGTCCTCTTACTTTCTC AATCTGACTTC	construction pVS5
vsp256_coaA_ovpPMA1_fw	GAAAGATTTAATTATCAAACAATATCAATATGTCTATT AAGGAACAAACC	construction pVS5
vsp257_tVMA2_ovtNAT5_rev	AATCACGAACTTTCATATTCAGGATCCGCGGCCGCGC TGATGTTCTTCGAGACCGGG	construction pVS5
vsp258_tVMA2_ovcoaA_fw	GAAGTCAGATTGAGAAAGTAAGAGGACGGTTGCTGA AGAAAAAGGC	construction pVS5
vsp259_tNAT5_ovCAB2_rev	ACTAAGTTGGCTACCAAGTAACAGATGGCTGAAAAA TTTTTTTTTTTTTTTTGG	construction pVS5
vsp260_tNAT5_ovtVMA2_fw	TCGAAGAACATCAGCGCGGCCGCGGATCCTGAATAT GAAAGTTCGTGATTCTCCTGC	construction pVS5
vsp261_CAB2_ovpGPM1_rev	CCAAACAAACACACATATTACAATAATGCCACCATTG CCAG	construction pVS5
vsp262_CAB2_ovtNAT5_fw	AAAAAAAAAAAAAATTTTTCAGCCATCTGTTACTTGG TAGCCAACTTAGTCTTAGAG	construction pVS5
vsp263_pGPM1_ovInt3'ADH6_rev	TACATTTATCAAGAGCTTGACAACACTAGTGGATCCC ACCCACACACAAGCTTC	construction pVS5
vsp264_pGPM1_ovCAB2_fw	TGTTCAAAACTGGCAATGGTGGCATTATTGTAATATG TGTGTTTGTTTGG	construction pVS5
vsp265_Int3'ADH6_ov2µ_rev	TTTATGCTTAAATGCGTGGCGCGCCACTAGTGGAACA TCCTCATCCGC	construction pVS5
vsp266_Int3'ADH6_ovpGPM1_fw	CTTGTGTGTGGGTGGGATCCACTAGTGTTGTCAAGCT CTTGATAAATGTAGC	construction pVS5

vsp269_pGPD2_fw	GGAACATCCGAGCACCCGCGCC	verification chromosomal integration pVS6
vsp270_tGPD2_rev	GGCGGCATCGAAATCTTCTTCTTGCCC	verification chromosomal integration pVS6
vsp271_seq43_tPRC1	GCAAAATTGTAGAAGCAGCTC	sequencing pVS5
vsp272_seq44_FEN2	TCTTTCTTGCAAATCAGCG	sequencing pVS5
vsp273_seq45_FEN2	GAACAACCATCTCCAACC	sequencing pVS5
vsp274_seq46_FEN2	TGATAGACTTAGATTCCTTCATC	sequencing pVS5
vsp275_seq47_tADH1	ATGAGTTGATGAATCTCGG	sequencing pVS5
vsp276_seq48_pPMA1	TTCAAATGTCCTATCATTATCGTC	sequencing pVS5
vsp277_seq49_coaA	TGGTAAGTCTACCACCGC	sequencing pVS5
vsp278_seq50_coaA	GAGCTTCTTTGATCTTGACC	sequencing pVS5
vsp279_seq51_tNAT5	GCTCTTAGACGTATATATTTCATC	sequencing pVS5
vsp280_seq52_CAB2	CAAGTATTGGTTGACGGTGG	sequencing pVS5
vsp281_seq53_pGPM1	ACAAAAAAAATTACAAAGGAAGG	sequencing pVS5
vsp282_seq54_Int3'ADH6	ACGTTCATAGGGTATCGG	sequencing pVS5
vsp283_loxP_ovMet25_rev	AGGGATTCGAACCCTTGCATCGCGGCCGCCCTAGGG ATCTGATATCACCTAATAACTTCG	construction coaA with pMet25
vsp284_Met25_ovloxP_fw	TTAGGTGATATCAGATCCCTAGGGCGGCCGCGATGC AAGGGTTCGAATCC	construction coaA with pMet25
vsp285_Met25_ovcoaA_rev	TCATCAAGGTTTGTTCCTTAATAGACATTCTAGAGTAT GGATGGGGGTAATAG	construction coaA with pMet25
vsp286_coaA_ovMet25_fw	AATTCTATTACCCCCATCCATACTCTAGAATGTCTATT AAGGAACAAACC	construction coaA with pMet25
vsp287_tVMA2_ovtNAT5_rev	ATTTATCAAGAGCTTGACAACACTAGTGCGGCCGCGC TGATGTTCTTCGAGACCGGG	construction pVS5-variants
vsp288_Int3'ADH6_ovtVMA2_fw	TCGAAGAACATCAGCGCGGCCGCACTAGTGTTGTCA AGCTCTTGATAAATGTAGC	construction pVS5-variants
vsp289_pFBA1_ovpRS62N_rev	AAAACGCCAGCAACGCGGCCTTTTTACGGTTCGGCGA ATTGTGGGTCATTACGTAAATAATGATAGG	construction pVS5-variants
vsp290_tPRC1_ovpRS62N_fw	GGTGTGAAATACCGCACAGATGCGTAAGGAGAAAAT ACCGCATATTGTACTTACAAACTCGGTAGTTGG	construction pVS5-variants
vsp291_tVMA2_ovpRS62N_rev	GGAAAAACGCCAGCAACGCGGCCTTTTTACGGTTCG GCGAATTGGCTGATGTTCTTCGAGACCGGG	construction pVS5-variants
vsp292_Met25_ovpRS62N_fw	GGTGTGAAATACCGCACAGATGCGTAAGGAGAAAAT ACCGCATGATGCAAGGGTTCGAATCC	construction pVS5-variants
vsp293_pPYK1_ovtdTer_rev	GTTGTTTCTAACCATTGGCTTAACAATCATTGTGATGA TGTTTTATTTGTTTTGATTG	construction pVS6/7/8 (tdTer)
vsp294_tdTer_ovpPYK1_fw	ACAAATAAAACATCATCACAATGATTGTTAAGCCAAT GGTTAGAAACAAC	construction pVS6/7/8 (tdTer)
vsp295_tdTer_ovtIDP1_rev	ATTGGGCTACGTAAATTCGATTAAATTCTGTCGAATCT TTCAACTTCAGC	construction pVS6/7/8 (tdTer)
vsp296_tIDP1_ovtdTer_fw	GCTGAAGTTGAAAGATTCGACAGAATTTAATCGAATT TACGTAGCCCAATCTAC	construction pVS6/7/8 (tdTer)
vsp297_pADH1_ovpTDH3_rev	GCCAGGAATAAACTGTGTTTAAACGCCGGCAAGTCC AATGCTAGTAGAGAAGG	construction pVS6/7/8 (EutE)
vsp298_pTDH3_ovpADH1_fw	CTCTACTAGCATTGGACTTGCCGGCGTTTAAACACAG TTTATTCCTGGCATCCAC	construction pVS6/7/8 (EutE)
vsp299_pTDH3_ovEutE_rev	TTGTTCAATATCTTGTTGGTTCATTTTGTTTGTTTATGT GTGTTTATTCG	construction pVS6/7/8 (EutE)
vsp300_EutE_ovpTDH3_fw	TCGAATAAACACACATAAACAAACAAAATGAACCAAC AAGATATTGAACAAGTTG	construction pVS6/7/8 (EutE)
vsp301_EutE_ovtRPL41B_rev	GAACTTAACGATTTGCTCTCAATCCGCTTAAACAATTC TGAAAGCATCGACC	construction pVS6/7/8 (EutE)
vsp302_tRPL41B_ovEutE_fw	TGGTCGATGCTTTCAGAATTGTTTAAGCGGATTGAGA GCAAATCG	construction pVS6/7/8 (EutE)
vsp303_tRPL41B_ovInt3'GPD2_rev	AGGGGGGGGGAGAGTGTGGCGCCGTTTAAACAGGG TAATATAATAAAGCCCTTCG	construction pVS6/7/8 (EutE)

vsp304_Int3'GPD2_ovtRPL41B_fw	GAAGGGCTTTATTATATTACCCTGTTTAAACGGCGCC ACACTCTCCCCCCCCCTC	construction pVS6/7/8 (EutE)
vsp305_tRPL3_ovBdhB_rev	TTGCAAATTTTCAAGAAGTCTGTTTAAGAAGTTTTGTT AGAAAATAAATC	construction pVS6/7/8 (BdhB)
vsp306_BdhB_ovtRPL3_fw	AAATGATTTATTTTCTAACAAAACTTCTTAAACAGACT TCTTGAAAATTTGC	construction pVS6/7/8 (BdhB)
vsp307_BdhB_ovpADH1_rev	CAAGCATACAATCAACTATCTCATATACAATGGTTGA TTTCGAATACTCTATTCC	construction pVS6/7/8 (BdhB)
vsp308_pADH1_ovBdhB_fw	GGAATAGAGTATTCGAAATCAACCATTGTATATGAGA TAGTTGATTGTATGC	construction pVS6/7/8 (BdhB)
vsp309_tVMA16_ovThlA_rev	GGTACCGCTATTTTGTTGGAAAAGTGTTAAGCGCTCA AACCAGGCTTTTC	construction pVS6/7/8 (ThlA)
vsp310_ThlA_ovtVMA2_fw	AGAAAAGCCTGGTTTGAGCGCTTAACACTTTTCCAAC AAAATAGCGG	construction pVS6/7/8 (ThlA)
vsp311_ThlA_ovpHXT7_rev	CAAAAAGTTTTTTTAATTTTAATCAAAAAATGAAGGA AGTTGTTATTGCTTCTGC	construction pVS6/7/8 (ThlA)
vsp312_pHXT7_ovThlA_fw	AGCAATAACAACTTCCTTCATTTTTTGATTAAAATTAA AAAAACTTTTTG	construction pVS6/7/8 (ThlA)
vsp313_seq55_tdTer	CCGTCTTGAAGCCATTCGG	sequencing pVS6/7/8
vsp314_seq56_tdTer	TTACAGACACGACTTCTTGGC	sequencing pVS6/7/8
vsp315_seq57_pTDH3	CAACTACAGAGAACAGGGGC	sequencing pVS6/7/8
vsp316_seq58_eutE	GGTGACAACGGTTTGACC	sequencing pVS6/7/8
vsp317_seq59_eutE	GCTGAACACCCATTCGC	sequencing pVS6/7/8
vsp318_seq60_BdhB	TCCAAAGCAATACCACCG	sequencing pVS6/7/8
vsp319_seq61_BdhB	CGTACTTCTTCAATTCTCTACCC	sequencing pVS6/7/8
vsp320_seq62_ThlA	ATCTTGGGTGTTCATCGG	sequencing pVS6/7/8
vsp321_CEN6ARS4_ovAmp_rev	GGTTCCGCGCACATTTCCCCGAAAAGTGCCACCTGAG GCCTGTCGACGGACGGATCGCTTGCC	pVS6/7/8 with CEN6ARS4 instead of 2µ
vsp322_CEN6ARS4_ovGPD2_fw	CGCTATGTTAGTCACTACTGACGGCTCTCCAGTCAGG CGCCGTCGACGGTCCTTTTCATCACGTGC	pVS6/7/8 with CEN6ARS4 instead of 2µ
vsp323_tACS1_rev	TTATAATGAGCAAGTCGATACAAGGACTGCCC	Δacs1 deletion cassette
vsp324_pACS1_fw	TCCTCCCAGAAAAACAATCTGTTTATTACCCG	Δacs1 deletion cassette
vsp325_tACS1_rev	CGTAAATCCTAGTGCATAAAAAGGCAG	verification of Δacs1 deletion cassette
vsp326_pACS1_fw	TAGTGTCGTTTGTGTCAGAGGAACG	verification of Δacs1 deletion cassette
vsp327_tSFA1_rev	AGTGGGTCGCCGATAATTATAGCGG	verification chromosomal integration of pVS4
vsp328_pSFA1_fw	GAATAAGTCCTGGTTCCAGGCAAAACC	verification chromosomal integration of pVS4
vsp339_SFA1_rev	CACCAGCGGCAGCCACACC	test-PCR within SFA1
vsp340_SFA1_fw	GGGCCACGAAGGAGCCGG	test-PCR within SFA1
vsp341_FEN2_ovMet25_rev	TTACCCCCATCCATACTCTAGAATGATGAAGGAATCT AAGTCTATCACTC	construction pVS5_5+5_6
vsp342_Met25_ovFEN2_fw	TGAGTGATAGACTTAGATTCCTTCATCATTCTAGAGTA TGGATGGGGGTAATAG	construction pVS5_5+5_6
vsp343_Met25_ovloxP_rev	TTATATTAAGGGTTGTCGCCTAGGGTTTAAACGATGC AAGGGTTCGAATCC	construction pVS5_5+5_6
vsp344_loxP_ovMet25_fw	GGATTCGAACCCTTGCATCGTTTAAACCCTAGGCGAC AACCCTTAATATAACTTCG	construction pVS5_5+5_6
vsp345_tVMA2_ovInt3'ADH6_rev	TTATCAAGAGCTTGACAACACTAGTGCGGCCGCGCTG ATGTTCTTCGAGACCGGG	construction pVS5_5+5_6
vsp346_Int3'ADH6_ovtVMA2_fw	GTCTCGAAGAACATCAGCGCGGCCGCACTAGTGTTGT CAAGCTCTTGATAAATGTAGC	construction pVS5_5+5_6
vsp347_tVMA16_ovNphT7_rev	GCTGCTTCTTTCGCTTTGATCGAATGGTAAGCGCTCA AACCAGGCTTTTC	construction pVS9
vsp348_NphT7_ovtVMA16_fw	AAAACGGAAAAGAAAAGCCTGGTTTGAGCGCTTACC ATTCGATCAAAGCG	construction pVS9

vsp349_NphT7_ovpHXT7_rev	AACAAAAAGTTTTTTTAATTTTAATCAAAAAATGACCG ACGTCAGATTCAG	construction pVS9
vsp350_pHXT7_ovNphT7_fw	TCTGAATCTGACGTCGGTCATTTTTTGATTAAAATTAA AAAAACTTTTTG	construction pVS9
vsp351_pPYK1_ovCcr_rev	TCCAAGATGTCCTTAACGGTCATTGTGATGATGTTTTA TTTGTTTTGATTG	construction pVS10
vsp352_ccr_ovpPYK1_fw	CAATCAAAACAAATAAAACATCATCACAATGACCGTT AAGGACATCTTGG	construction pVS10
vsp353_ccr_ovtIDP1_rev	TAGATTGGGCTACGTAAATTCGATTAGACGTTTCTGA ATCTGTTGATAGC	construction pVS10
vsp354_tIDP1_ovCcr_fw	CGCTATCAACAGATTCAGAAACGTCTAATCGAATTTA CGTAGCCCAATCTAC	construction pVS10
vsp355_seq63_NphT7	AGTCAGCACCGATGACC	sequencing pVS9/10
vsp356_seq64_ccr	CAAAGCACTTGACCTGGG	sequencing pVS9/10
vsp357_seq65_ccr	AGAAACCGGTCAAGCTGC	sequencing pVS9/10
vsp358_loxP_ovInt'5ADH6_fw	AAAAGAAAAGTGAAAGCCTGCAGGATCCTAGGCGAC AACCCTTAATATAACTTCG	construction pVS5_5
vsp359_Int'ADH6_ovloxP_rev	TTATATTAAGGGTTGTCGCCTAGGATCCTGCAGGCTT TCACTTTTCTTTTTCTCC	construction pVS5_5
vsp360_CEN6ARS4_ovInt3'SFA1_fw	TGTTGCCATCGATAACGGTGGTGACATAAATTCCAGG CGCCGCCGGCGGTCCTTTTCATCACGTGC	construction pVS4 with CEN6ARS4 instead of 2µ
vsp361_CEN6ARS4_ovAmpColE1_rev	TTCCGCGCACATTTCCCCGAAAAGTGCCACCTGGGCG CGCCGCCGGCGGACGGATCGCTTGCC	construction pVS4 with CEN6ARS4 instead of 2µ
vsp362_pADH6_fw	TTTAATTTGTATTGCTTCCGGTTTGCGTTGG	verification chromosomal integration pVS5.5
vsp363_tADH6_rev	ACAACAAAGGCTTCTCCATCAACTTCCCC	verification chromosomal integration pVS5.5
vsp364_ADH6_fw	GACATTAAGATCGAAGCATGTGGTGTCTGCGG	test-PCR within ADH6
vsp365_ADH6_rev	CCTTTCGAAGGCTTCATGGACGCCGG	test-PCR within ADH6

Table S4: Statistical analysis of n-butanol production

A t-test was performed (unpaired, two-tailed, confidence: 95%) with program GraphPad Prism 5. The titers of figure 5 and the yields of figure S4 were compared (strain 1 with strain 2).

Strain 1	Strain 2	p-value of titer	p-value of yield
WT + pVS6	WT + pVS6 + pVS5_4	0.0479	0.1518
WT + pVS6 + pVS5_4	WT + pVS6 + pVS5_4 + 25 µM pantothenate	0.0004	0.0012
VSY0 + pVS6	VSY0 + pVS6+ pVS5_4	0.0008	0.0004
VSY0 + pVS6+ pVS5_4	VSY0 + pVS6+ pVS5_4 + 25 µM pantothenate	0.0731	0.0085
VSY8 + pVS6	VSY8 + pVS6+pVS5_4	0.0002	<0.0001
VSY8 + pVS6+pVS5_4	VSY8 + pVS6+pVS5_4 + 25 µM pantothenate	0.0306	0.3894
VSY10	VSY10 + 25 µM pantothenate	0.1596	0.0092

References

1. Becker J, Boles E. A modified *Saccharomyces cerevisiae* strain that consumes L-arabinose and produces ethanol. Appl Environ Microbiol. 2003;69:4144–4150.
2. Tornow J, Santangelo GM. Efficient expression of the *Saccharomyces cerevisiae* glycolytic gene *ADH1* is dependent upon a cis -acting regulatory element (UASRPG) found initially in genes encoding ribosomal proteins. Gene. 1990;90:79–85.
3. Capieaux E, Vignais ML, Sentenac A, Goffeau A. (1989). The yeast H+-ATPase gene is controlled by the promoter binding factor TUF. J Biol Chem. 1989;264:7437–7446.
4. Rodicio R, Heinisch JJ, Hollenberg CP. Transcriptional control of yeast phosphoglycerate mutase-encoding gene. Gene. 1993;125:125–133.
